# Iodine Deficiency, Maternal Hypothyroxinemia and Endocrine Disrupters Affecting Fetal Brain Development: A Scoping Review

**DOI:** 10.3390/nu15102249

**Published:** 2023-05-09

**Authors:** Rolf Grossklaus, Klaus-Peter Liesenkötter, Klaus Doubek, Henry Völzke, Roland Gaertner

**Affiliations:** 1 Department of Food Safety, Federal Institute for Risk Assessment, D-10589 Berlin, Germany; rgrossi@web.de; 2 Endokrinologikum, Center for Hormonal and Metabolic Diseases, D-10117 Berlin, Germany; kp.liesenkoetter@posteo.de; 3 Professional Association of Gynecologists, D-80337 Munich, Germany; 4 Study of Health in Pomerania/Clinical-Epidemiological Research, Institute for Community Medicine, University Medicine Greifswald, D-17475 Greifswald, Germany; voelzke@uni-greifswald.de; 5 Medical Clinic IV, University of Munich, D-80336 Munich, Germany

**Keywords:** iodine deficiency, pregnancy, hypothyroxinemia, neurocognitive development, endocrine disruptors, brain development

## Abstract

This scoping review critically discusses the publications of the last 30 years on the impact of mild to moderate iodine deficiency and the additional impact of endocrine disrupters during pregnancy on embryonal/fetal brain development. An asymptomatic mild to moderate iodine deficiency and/or isolated maternal hypothyroxinemia might affect the development of the embryonal/fetal brain. There is sufficient evidence underlining the importance of an adequate iodine supply for all women of childbearing age in order to prevent negative mental and social consequences for their children. An additional threat to the thyroid hormone system is the ubiquitous exposure to endocrine disrupters, which might exacerbate the effects of iodine deficiency in pregnant women on the neurocognitive development of their offspring. Ensuring adequate iodine intake is therefore essential not only for healthy fetal and neonatal development in general, but it might also extenuate the effects of endocrine disruptors. Individual iodine supplementation of women of childbearing age living in areas with mild to moderate iodine deficiency is mandatory as long as worldwide universal salt iodization does not guarantee an adequate iodine supply. There is an urgent need for detailed strategies to identify and reduce exposure to endocrine disrupters according to the “precautional principle”.

## 1. Introduction

Thyroid hormones are particularly important for normal embryonal/fetal and early postnatal neurocognitive development. Depending on the severity, duration and timing of iodine deficiency in certain life stages, iodine deficiency disorders (IDDs) may be associated with physical, neurological and intellectual deficits in humans. Severe iodine deficiency during pregnancy can lead to a number of adverse effects on maternal and child health, including goiter, hypothyroidism, stillbirth, increased neonatal mortality, neurological damage, and mental impairment [1]. In addition, global exposure to endocrine-disrupting chemicals (EDC) is increasing [2,3,4]. Exposure to these chemicals in the presence of inadequate iodine supply might be additionally harmful to the embryonal/fetal and neonatal brain development, growth, differentiation, as well as metabolic processes in adulthood [5,6,7,8,9,10,11,12]. 

Both iodine deficiency and exposure to EDCs have a negative impact on general health and on socio-economic systems. Annual costs for seven categories of EDCs with the highest causality have been estimated to be at least €33.1 billion in Europe. The largest proportion of costs is related to the loss of IQ points and neurocognitive diseases [13,14,15,16,17]. In addition, there is growing evidence that exposure to EDCs, including air pollution, affects not only the development of brain function [10,18,19,20,21] but also the outcomes of pregnancy and childbirth [22,23,24,25,26].

The “endemic goiter” has long been a synonym for iodine deficiency, and the aim has always been to prevent the enlargement of the thyroid gland and overt thyroid dysfunction. Within the last decades, however, the consequences of mild to moderate iodine deficiency on the cognitive development of the embryo/fetus have also come into focus [27]. 

Epidemiological and experimental studies of mild to moderate iodine deficiency over the past two decades have shown that embryonal/fetal brain development can be impaired not only in hypothyroid mothers but also in hypothyroxinemic mothers in the early stages of pregnancy [28,29,30,31,32]. Subtle changes in fetal brain development were observed even with maternal thyroid hormone levels within the lower reference range, although the results are not homogeneous, and, therefore, isolated maternal hypothyroxinemia (IMH) is not yet generally accepted as an independent thyroid disease. Instead, it is assumed to be the result of iodine deficiency and/or EDC contamination or of other factors, as extensively summarized and discussed by others [6,33,34,35].

The aim of this scoping review is to identify gaps in knowledge about the effects of mild and moderate iodine deficiency and additional environmental influences such as EDCs and air pollution during pregnancy on the cognitive and psychosocial development of the offspring and how significant IMH might be. Above all, the results of systematic reviews should be used to present the need for a safe and secure iodine supply to political decision-makers.

## 2. Materials and Methods

**Figure 1:**
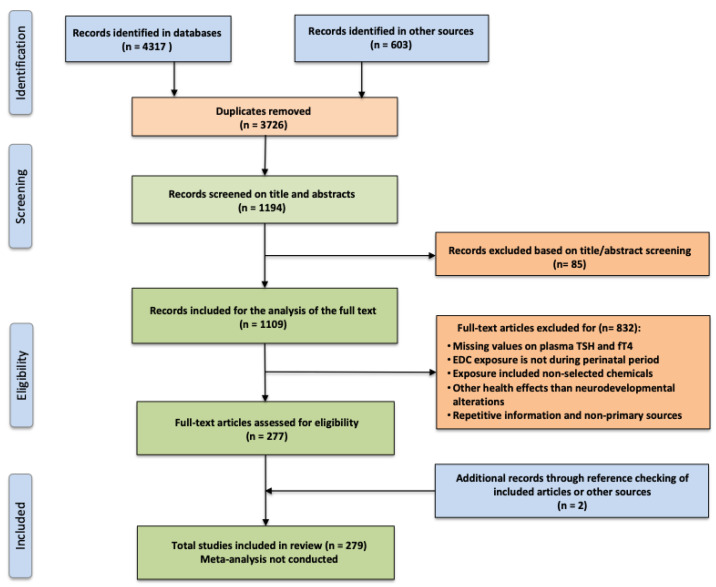
The PRISMA diagram showing the search strategy and inclusion/exclusion criteria at each step.

This study is a scoping review in accordance with the PRISMA Extension for Scoping Reviews (PRISMA-ScR) statement [36]. The study selection process is documented in Figure 1. Literature searching was performed in PubMed, Medline, Cochrane, Web of Science, Google Scholar, World Health Organization (www.who.int/en/, accessed on 1 April 2022), and Iodine Global Network (https://www.ign.org, accessed on 1 April 2022) databases between January 1990 and April 2022. Keywords were iodine, pregnancy, thyroid hormone, thyroid diseases, endocrine disruptors, hypothyroxinemia and subclinical hypothyroidism, searched for in combination using AND and OR operators. Inclusion criteria were observational and interventional studies, including reviews and meta-analyses, which assessed the iodine status of pregnant women in Europe; assessed thyroid hormone concentrations (fT4, TSH) during the first trimester of pregnancy; iodine supplementation before and during pregnancy and perinatal exposure of certain EDCs, which were limited to potential thyroid-disrupting chemicals (TDCs) such as organochlorine pesticides (OCPs), polychlorinated biphenyl compounds (PCBs, OH-PCBs), perchlorate, thiocyanate, nitrate, phthalates, genistein, 4-nonylphenol (NP), benzophenone 2 (BP2), amitrole, polybrominated diphenylethers (PBDEs), triclosan and bisphenols. 

The initial search retrieved a total of 4920 articles. All duplicate papers were double-checked and excluded. Titles and abstracts were screened for relevance independently by two reviewers (RGr and KPL), with any disagreements being resolved by discussion and involvement of a third reviewer (RGa) where necessary. The full text of potentially relevant papers was retrieved and screened in the same way using the prespecified inclusion and exclusion criteria. Additional records were identified from the reference lists of retrieved publications or through other sources. A total of 279 articles remained eligible for inclusion in this study. The results were discussed with all authors during virtual meetings.

## 3. Results and Discussion

### 3.1. Iodine Requirements and Iodine Status of Pregnant Women in Europe

Pregnant women have an approximately 50% higher iodine requirement compared to non-pregnant women due to increased thyroid hormone production, increased renal iodine clearance and transplacental transmission of iodine to the fetus [37]. Accordingly, the recommendation for mean iodine intake during pregnancy is 150–249 µg/day [38].

The WHO recommends four possible markers to evaluate the iodine intake of a defined population: urinary iodine concentration (UIC), the serum concentration of thyroid stimulating hormone (TSH), serum concentration of thyroglobulin (Tg) and the thyroid volume [39], but not free thyroxine (fT4). Mean UIC is used in most epidemiological studies because of the easy practicability that testing involves. A mean UIC of 250 µg/L during pregnancy is assumed as an adequate iodine supply [39,40]. However, no epidemiological studies so far have compared the four parameters to find the best method.

It is estimated that worldwide, approximately 54% of women have insufficient iodine supply during pregnancy [41]. Within Europe, this percentage is even higher; more than 70% (*n* = 21) of the 29 European countries have an insufficient iodine intake (Table 1) [42,43,44,45,46,47,48,49,50,51,52,53,54,55,56].

The reason is that most European women of childbearing age live in countries with voluntary household salt iodization. As a result, the median UIC in these countries is below 100 μg/L [57]. Recently, national representative data from children and adolescents in Germany revealed a mean UIC of 89 µg/L [55,58]. Iodine intake has clearly decreased in the last decade compared to the baseline survey (2003–2006). The cause might be that the use of iodized salt in artisanal and industrially produced foods has declined, and vegetarian and vegan diets have been attracting more people, particularly young people [59]. 

A median UIC > 150 μg/L was reported in only a few EU states with mandatory universal salt iodization programs, such as Bulgaria or Romania (see Table 1). In Poland and Italy, iodization of table salt is mandatory but not allowed in processed foods, with the exception of formulations for toddlers [60,61,62]. Belgium, Denmark and the Netherlands have introduced the mandatory use of iodized salt in bread. However, this strategy does not seem to meet women’s higher iodine requirements during pregnancy [63,64]. Additional iodine supplementation for women of childbearing age might be necessary in most European countries [40,65,66]. The trend of a re-emergence of iodine deficiency among vulnerable groups, such as reproductive-age women in most European countries, appears to mirror the trends in other industrialized nations [67].

### 3.2. Isolated Maternal Hypothyroxinemia (IMH)

#### Definition, Prevalence and Causes

The first study described IMH during pregnancy as the free maternal serum thyroxine concentration (fT4) being below the 10th percentile with a TSH value in the reference range [68]. However, there are significant differences in the definition of IMH (fT4 below 10th or 2.5th percentile) in later studies that make the interpretation of the data difficult [69,70,71]. The prevalence of IMH varies between 1% and 25%, depending mainly on the diagnostic criteria, the trimester of pregnancy or the method of fT4 measurement. In countries with iodine deficiency, the frequency of IMH is expected to be higher [33,72]. IMH might be due primarily to mild or moderate iodine intake, which is associated with an increased release of triiodothyronine (T3) and decreased T4 to save iodine. As only fT4 is actively transported through the placenta in the first 4 months, IMH might affect embryonic brain development before the fetal thyroid function starts [73]. 

Prenatal exposure of maternal and embryonal/fetal thyroid to EDCs might additionally influence the brain development of the offspring [6,7,74]. Ethnicity, mother’s age, parity, pre-pregnancy body mass index and vitamin D deficiency (as potential causative factors) are also associated with IMH [33,75,76,77,78,79,80]. It should also be considered that the shortage of trace elements beyond iodine, e.g., selenium and iron, are particularly important for thyroid function and should also be viewed as risk factors of IMH [81,82,83].

ATA guidelines do not recommend fT4 analysis in pregnancy but only total T4 [69] measurement. The recommended method to assess fT4 in pregnancy would be dialysis or ultrafiltrate of serum samples employing liquid chromatography-tandem mass spectrometry (LC/MS/MS) [69,70,84]. This, however, has never been done in epidemiologic studies. 

The most recent clinical guidelines, therefore, regard population-based, trimester-specific reference ranges for serum TSH and T4 levels in a local, euthyroid, pregnant population as the gold standard [69,85]. 

### 3.3. Prenatal Brain Development, Timing of Thyroid Hormone Action and Identification of Specific Thyroid-Related Modes-of-Actions (MoAs) in Connection with EDCs

**Figure 2:**
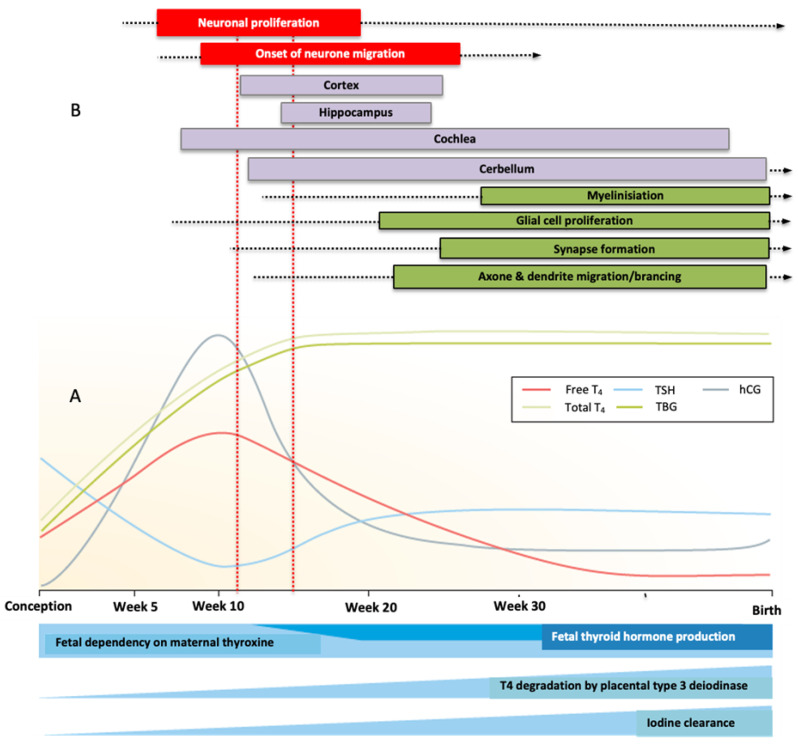
Changes in thyroid physiology during pregnancy (**A**) and the relationship between thyroid hormone activity and brain development (**B**) (adapted from [88,89]. (**A**) shows the main changes in thyroid physiology during pregnancy. An increase in thyroxine-binding globulin (TBG), an increase in thyroxine consumption by the fetus, and an increase in placenta type 3 deiodinase expression require upregulation of thyroid hormone production in order to maintain sufficient availability of thyroid hormones. These upregulations are largely mediated by increased thyroid stimulation by human chorionic gonadotropin (hCG), which ultimately leads to a net increase in free T4 concentration and a subsequent decrease in TSH concentration. (**B**) shows the relationship between thyroid hormone activity and brain development. Note the time window (between the two red dotted lines) in which a drop in maternal thyroid hormone (fT4) has a particular impact on neural proliferation and migration, and development of the inner ear. For further explanations, see the text.

The embryonal and early fetal brain development depends on the maternal T4 (Figure 2) [74,86,87,88,89,90]. Only fT4 enters the embryonal/fetal blood–brain barrier. Exposure to some ECDs has been shown to interfere with the thyroid system at multiple sites affecting the developing brain, for example, by inhibiting deiodinase activities as well as with transport mechanisms like transthyretin (TTR) in the placenta, in the embryo/fetus [6,9,11,74]. 

Identifying this early, critical period can have direct clinical implications for risk assessment and the window of opportunity for treatment (s. Figure 2). During this critical phase of development, reduced maternal placental fT4 transfer most likely has the greatest impact on a child’s neurological development [91,92,93,94,95]. Brain MRI imaging enables objective measurements of brain development and provides detailed information about brain structures. Imaging data provide information on neurogenic processes during certain stages of fetal brain development [96,97].

The results of part of the longitudinal Generation R study in Rotterdam, including 1981 mother-child pairs, indicate a “fetal programming effect”. Measurements of maternal TSH and fT4 in early or middle pregnancy (≤18 weeks) were compared with MRI data from the brains of children aged 10 years. It was found that there was an inverted U-shaped association of maternal TSH with the total volume of gray matter in the offspring and with the volume of cortical gray matter. It was also shown for the first time that this association with a later occurrence of neurodevelopmental disorders is more obvious when thyroid function is measured before the 14th week of pregnancy. Both low and high maternal thyroid function, particularly in early pregnancy, were shown to be associated with smaller child total grey matter and cortical volume [98,99]. However, no causal relationship between maternal thyroid function and morphological brain changes in the child has yet been conclusively clarified. Further research is warranted to determine how MRI, as a morphometric method, can help to assess the effects of maternal thyroid function on the child’s cognitive function [100,101].

There is increasing evidence that TDCs also interfere with several intracellular thyroid hormone actions and brain development [6,9,11,102,103,104,105,106,107,108]. Obviously, iodine deficiency might promote these deleterious effects and thus deregulate transcription and thyroid hormone-induced epigenetic effects on target genes [109]. The urgency of this issue is the coincidence of the still prevailing inadequate iodine supply and continuously increasing exposure of humans to TDCs [6,22,110,111,112]. 

TDCs affect pregnancy not only by acting as hormonal agonists/antagonists but also indirectly by disrupting maternal, placental, and fetal homeostasis. This might be mediated by an impact on oxidative stress, the hormonal milieu, metabolomic profile and microbiome [22,113,114,115,116,117,118,119,120]. It is believed that the adverse health effects in offspring caused by EDCs, including air pollution, can be caused by two mechanisms: first, directly through the placenta and thus to the fetal circulation and/or second, indirectly through oxidative stress of the placenta, inducing inflammation and epigenetic changes in the placenta as well as in the offspring [10,121,122,123,124,125,126]. 

Epidemiological studies on physiological fluids collected in pregnant women (blood, serum, urine, amniotic fluid, placenta) show that combined exposure to EDCs (“cocktails”) is commonplace and widespread [127,128,129]. It is unlikely that safe levels of EDC contamination can be defined because of the diverse actions of EDCs like low dose effects, possible non-linear dose responses, cumulative effects often expected from combined exposure and trans-generational effects with different effects during vulnerable exposure windows [16,130,131,132,133,134].

Some of the TDCs’ effects on TH metabolism are well characterized and summarized in Table 2 [135,136,137,138,139,140,141,142,143,144,145,146,147,148,149,150,151,152,153,154,155,156,157,158,159,160,161,162,163,164,165,166].

Ortho-substituted polychlorinated biphenyl (PCP) congeners (95 or 101) decrease pituitary response to thyrotropin-releasing hormones [167]. Perchlorate, thiocyanate and nitrate competitively inhibit the iodide uptake by the NIS. This might also be true for benzophenone 2 [102].

Animal studies have indicated that phthalates alter thyroid signaling through a number of potential mechanisms, including interference with the TSH receptor, binding to transport proteins, interfering with the hypothalamic-pituitary-thyroid axis, and changing NIS-mediated iodide uptake, iodothyronine deiodinases, or hepatic enzymes [102,168].

PCPs and their metabolites, as well as polybrominated diphenyl ethers (PBDEs), also bind TTR and displace T4 [7,102,104,105]. TTR has been proposed as being of importance by transferring thyroid hormones across the blood–brain barrier as well as via placenta to the fetal compartment. Another mechanism for the decrease in serumT4 concentrations in both adults and neonates may be the ability of PCBs to induce hepatic microsomal enzymes leading to biliary excretion and elimination of the thyroid hormones. Such changes could be particularly adverse during early pregnancy and even have long-term effects on the brain, as demonstrated in Table 2 [3,153,154,155,156,157,169,170].

Bisphenols, including BPA, can interfere with thyroid hormone synthesis, transport and metabolism. The main mechanism of action is thought to be the binding of BPA to TR and interference with thyroid hormone [166]. Human data during pregnancy substantiate experimental findings suggesting that BPA could potentially affect thyroid function and deiodinase activities in early gestation (6–14 weeks). BPA was associated with a lower ratio of both fT4/fT3 and TT4/TT3 (total thyroxine/total triiodothyronine), as well as a lower TT4 concentration [171]. BPA at environmentally-relevant doses also has epigenetic actions that can lead to heritable changes in gene expression. BPA might alter DNA methylation quite early in embryonic development and may impact the chromatin state of germ cells [172].

Whatever the level at which the disruption occurs, it can result in decreased T3 binding to nuclear TRs. This might then modulate transcriptional activity and induce epigenetic disruption [9,173,174,175,176,177]. Changes in TH availability—and thus in DNA methylation rates—during organogenesis and developmental transitions can not only increase the risk of a low IQ but also lead to other long-term offspring outcomes such as cardiometabolic, neurodevelopment and behavioral defects [9,92,109,175,178].

### 3.4. Mild and Moderate Iodine Deficiency and Its Consequences

In contrast to severe iodine deficiency, a mild to moderate intrauterine iodine deficiency has more subtle but nonetheless important long-term cognitive and psychosocial consequences for the offspring [179]. 

In observational studies about adverse effects on cognitive development and behavioral disorders related to mild iodine deficiency, maternal blood samples were usually taken between the ninth and 13th week of pregnancy (Table 3). The neurological examinations of the offspring were carried out between the ages of 6 months and 16 years [95]. Overall, the study designs were very different. The differences relate to the criteria for the selection of mother-child pairs, to the reference values and ranges used to determine the different degrees of maternal hypothyroidism and/or hypothyroxinemia, and to the different tests for neurological development used (s. Table 3).

All studies, except one by Grau et al. [187], who examined the effects of low maternal fT4 levels towards the end of the first trimester, reported impairment of cognitive and motor development in exposed children [30,34,69,91,93,181,182,183,188,189,190]. With the progress of pregnancy, the correlation weakened gradually and disappeared until late pregnancy [32,186,191]. 

Two systematic reviews [192,193] and five meta-analyses [180,194,195,196,197] evaluated the association between maternal iodine deficiency and intellectual outcomes in the offspring. In general, these publications suggested an association between high TSH and/or low T4 levels in the serum of pregnant mothers and impaired neurological development and behavioral problems in the child.

Using the random effects model, two meta-analyses [180,195] revealed that subclinical hypothyroidism and hypothyroxinemia in mothers are associated with indicators of intellectual disability in the offspring (odds ratio [OR] 2.14, 95% confidence interval [CI] 1.20 to 3.83, *p* = 0.01 and OR 1.63, 95% CI) 1.03 to 2.56, *p* = 0.04) [180]. On the other hand, the results for behavioral disorders like ADHD or autism are inconsistent and require further studies [180,197]. The meta-analysis by Levie et al. [197], who evaluated data from three cohort studies together, found no clear indications for a connection between maternal TSH and fT4 up to the 18th week of pregnancy and ADHD in the offspring [197]. The systematic review by Drover et al. [193], which included 28 studies (5 of which looked at thyroid hormone levels in newborns), reported moderate evidence of such an association between IMH and ADHD.

Overall, none of the systematic reviews and meta-analyses showed clear cut-offs for high TSH and/or low fT4 levels in the serum of pregnant mothers, which indicates an increased risk of neurodevelopmental disorders in children. Such cut-offs could not be determined because the epidemiological studies were not designed to show quantitative limits (s. Table 3).

### 3.5. Influence of TDCs, Including Air Pollution on Embryonic/Fetal Neurodevelopment in Iodine-Deficient Areas

So far, studies investigating maternal hypothyroxinemia due to mild to moderate iodine deficiency have not considered additional prenatal exposure to TDCs (s. Table 2, right column). However, retrospective case-control and cohort and population studies linking TDC exposure with epidemiological data on thyroid hormone-related (dys-)functions provide clear evidence that the development of the embryonal/fetal and neonatal brain as well as growth, differentiation and metabolic processes are at risk of suffering adverse TDCs effects [6,198]. In recent years, there has been a significant increase in neurodevelopmental disorders, including autism and ADHD [2,12,199,200,201,202].

Public health concern exists for mildly iodide-deficient pregnant women who are exposed to perchlorate, thiocyanate, nitrate or other environmental antithyroid agents [4,8,11,16,203,204,205,206,207,208,209]. In a dose-response model between iodide and perchlorate exposure in food, it was shown that a low iodide intake of 75 μg/day and a perchlorate daily dose of 4.2 μg/kg are sufficient to induce hypothyroxinemia, while an adequate iodine intake of 250 μg/day a higher perchlorate daily dose of around 34 μg/kg is required [210]. Iodine supplementation would be sufficient to prevent the goitrogenic effects of perchlorate exposure at current regulatory limits among at-risk individuals [205]. Iodine deficiency could therefore deteriorate the effects of TDCs exposure, especially during pregnancy [4,8,11,16,104,105].

Certain phthalates, including di-(2-ethylhexyl) phthalate (DEHP) and di-n-butylphthalate (DnBP), have antithyroid activity occurring through several possible mechanisms, such as down-regulation of NIS and interacting with hormone synthesis-related proteins, deiodinases, TTR, receptors, and hepatic enzymes [6,9,211,212]. Because phthalates may have multiple and possibly overlapping targets in the HPT axis, sometimes acting as an agonist or antagonist, the outcome from a given phthalate blend may not be predictable. For example, it had been shown in the Norwegian mother, child and father cohort study (MoBa) that exposure to certain phthalates in pregnant women increased TT3 and FT3, but only when iodine intake was low (<150 µg/d), whereas in those women with high iodine intake (>150 µg/d), TSH increased, and TT4 and FT4 decreased [213].

Furthermore, epidemiologic evidence suggests that prenatal exposure to phthalates is associated with emotional and behavioral difficulties in children [9,145,146,147,148,149,214,215]. However, a recent literature review including 17 epidemiological studies reveals no clear pattern of association between maternal exposures to phthalates during pregnancy and offspring neurodevelopment. This, again, might be caused by inconsistent study protocols, test systems and confounders [216,217].

A prospective pregnancy and birth cohort study examined BPA interaction with thyroid hormones in pregnant women and newborns. Higher BPA exposure is associated with decreased TSH in umbilical cord serum in girls. BPAs have the greatest negative effects on girls born to mothers with iodine deficiency [218]. A birth cohort study in China showed that the concentration of BPA in urine in the prenatal period was associated with low TSH in overweight mothers, but there was no association with fT4, fT3 and TSH in umbilical cord serum [219]. The disturbance of thyroid hormone (TH) levels as a result of prenatal exposure to BPA may be associated with long-term neurobehavioral changes at a later age [220,221,222]. It should be noted that there are various kinds of test batteries for child neurodevelopmental assessment at different ages whose findings have been inconsistent among studies. In addition, the timing and number of exposure assessments have varied. However, ADHD symptoms, especially among boys, constantly suggested an association with both prenatal and concurrent exposure to BPA [223]. Although there is limited evidence on the adverse effects of prenatal and postnatal BPA exposures, pregnant women and young children should be protected from exposure based on a precautionary approach [224,225].

Furthermore, a pilot study suggests that 2,3′,4,4′,5-pentachlorobiphenyl (PCB 118) has a negative impact on neurocognitive development and probably reduces the benefits of iodine supplementation in areas with borderline iodine deficiency. Therefore, TDC exposure should be considered when designing studies on the benefits of iodine supplementation during pregnancy [226].

From observational studies, there is strong evidence that TDCs are disturbing the thyroid hormone metabolism (s. Table 2). This is reported for several classes of TDCs, for mother-child pairs, in case controls, smaller cohorts or larger epidemiological studies [6,102,135,136,137,138,139,140,141,142,143,144,145,146,147,148,149,150,151,152,153,154,155,156,157,158,159,160,161,162,163,164,165,166,167,168,169,170,171,172,173,174,175,176,177,178,179,180,181,182,183,184,185,186,187,188,189,190,191,192,193,194,195,196,197,198,199,200,201,202,203,204,205,206,207,208,209,210,211,212,213,214,215,216,217,218,219,220,221,222,223,224,225,226,227,228,229,230,231,232]. However, further research and long-term clinical studies are necessary to finally elaborate on the dose-related associations [6,168,227,229,233,234,235,236].

Air pollution is a leading risk factor for the global disease burden, but the negative effects of exposure to particulate matter <2.5 μm (PM_2.5_) during pregnancy have not been considered in the past [237,238,239]. However, there is growing evidence of the negative effects of exposure to burn-related air pollution on the neurological development of fetuses and childhood behavior [20,240,241,242]. Air pollution may interfere with maternal thyroid function during early pregnancy, as shown in cohort studies from four European cohorts [10] and in Shanghai [243]. A 10 mcg/m^3^ increase in PM_2.5_ exposure in both the first and second trimester was associated with 28% (OR = 1.28, 95% CI, 1.05–1.57) and 23% (OR = 1.23, 95% CI, 1.00–1.51) increases in the odds of maternal hypothyroxinemia, respectively [243]. However, both studies have some limitations. Neither the iodine concentration in the urine of the pregnant women [244] nor the exposure to other environmental chemicals was considered [245].

The available evidence suggests that intrauterine PM_2.5_ exposure can alter prenatal brain development through oxidative stress and systemic inflammation, leading to chronic neuroinflammation, microglial activation, and neuronal micturition disorder [18,168,246]. It has been shown that particulate matter exposure during fetal lifetime was associated with structural changes in the child’s cerebral cortex, as well as with impairment of essential executive functions, such as inhibitory control [247,248].

Studies that focused on exposures to air pollution, especially PM and NO_2_, during the prenatal period and the first years of life found associations with reduced psychomotor development [249,250] and impairment in cognitive development [19,251,252], as well as with autism-spectrum disorders [201,253,254,255]. However, these results could not be confirmed by others [20,21,256,257,258].

### 3.6. Prevention and Treatment of IMH

Since studies on the effects of IMH on cognitive and motor development, as well as on the risk of neuropsychiatric diseases in children, show a clear connection to early pregnancy; the central clinical question remains whether these complications can be prevented by early iodine supplementation or L-Thyroxine substitution [29,33].

Treatment of IMH or subclinical hypothyroidism with L-Thyroxine during early pregnancy revealed no benefit concerning the neurodevelopment of the children at the age of 6 and 9 years. However, L-Thyroxine supplementation started at the mean of the 12th week of pregnancy, which is too late [259,260]. This is why the ATA guidelines do not recommend L-Thyroxine supplementation [69]. However, based on new epidemiological data, ETA guidelines are considering L-Thyroxine supplementation during the first trimester rather than later [85]. The results of a recent study showed that early L-Thyroxine supplementation in women with TSH levels of >2.5 mU/L and fT4 < 7.5 pg/mL at or before the ninth gestational week (GW9) is safe and improves the progress of gestation. Whether the neurodevelopment of these offspring also improved, however, has not been studied so far. These data support the recommendation to adopt these cut-off levels for L-Thyroxine supplementation, which should be started as early as possible, ideally before the end of the first trimester of gestation, and TSH suppression should be avoided [261].

In regions with mild to moderate iodine deficiency, iodized salt intake, regularly used at least 24 months before pregnancy, can significantly improve maternal thyroid economy and reduce the risk of maternal thyroid insufficiency during pregnancy. This is probably due to a restoration of intrathyroidal iodine stores [262,263,264,265,266,267,268]. The importance of this finding is supported by the results of a large prospective cohort, including mothers and offspring. A positive association between preconception maternal iodine status and the cognitive function of the offspring at the age of 6–7 years could be demonstrated [265]. In contrast, meta-analyses of iodine supplementation starting during pregnancy found no effect on child neurodevelopment [196,264,269,270,271,272]. The lack of beneficial effects of iodine supplementation, typically after the first trimester, bypasses the critical period of development early in gestation.

There is some concern that over-the-counter iodine-containing supplements might contain high doses that temporarily disturb thyroid hormone production and/or release. Therefore, moderate iodine deficiency should be prevented already before conception [273,274]. Well-designed randomized controlled trials investigating a daily supplementation with 150–200 µg iodine in preconception, pregnancy, and lactation are underway to investigate children’s neuropsychological development [275,276,277,278]. Also, more data are needed to determine optimal and safe upper limits of iodine supplementation in pregnant women and assess the potential risks of chronic high iodine intake during pregnancy [270,279].

The Krakow Declaration of Iodine, published by the EU thyroid consortium and other organizations, raised major points about how iodine deficiency can be efficiently eradicated in Europe. It was demanded that (1) universal salt iodization should be harmonized across European countries, (2) regular monitoring and evaluation studies have to be established to continuously measure the benefits and potential harms of iodine fortification programs and (3) societal engagement is needed to warrant sustainability of IDD prevention programs [280].

## 4. Conclusions

A paradigm shift has occurred in assessing the epidemiology of IDD away from goiter and focusing on the iodine status of pregnant women. This is still insufficient in most European countries, including Germany. Mild to moderate iodine deficiency, as well as probable IMH in early pregnancy, might have long-term negative effects on the cognitive and psychosocial development of children. Population-based, trimester-specific reference ranges for serum TSH and fT4 levels need to be used for the diagnosis.

Effective iodine prophylaxis for negative effects on the cognitive and psychosocial development of children should be carried out preconceptually, as the adverse effects on the developing brain are caused by reduced availability of maternal fT4 in the first weeks of pregnancy. Effective prevention through iodine supplementation for risk groups, including women of childbearing age, should, therefore, take place before conception as long as there is no nationwide universal salt iodization.

Adequate iodine supply needs to be achieved globally as this may reduce some adverse effects of additional exposure to TDCs. According to the “precautionary principle” of reducing the risk even in the absence of causal evidence, measures should be taken to reduce the environmental impact of TDCs.

## Figures and Tables

**Table 1:** Iodine intake in the general population and in pregnant women in Europe.

Country	General Population ^a^				Pregnant Women ^b^			
	Median (UIC) (μg/L)	Date of Survey (N, S)	Population	Population Iodine Intake	Median (UIC) (μg/L)	Date of Survey (N, S)	Iodine Intake	Legislation Status ^e^ (Year)
Austria	111	2012 (N)	SAC (7–14)	Adequate	87	2009–2011 (S)	Insufficient	Mandatory (1999)
Belgium	113	2010/11 (N)	SAC (6–12)	Adequate	124	2010 (N)	Insufficient	Voluntary (2009)
Bulgaria	182	2008 (N)	SAC (7–11)	Adequate	165	2003 (N)	Adequate	Mandatory (2001)
Croatia	248	2009 (N)	SAC (7–11)	Adequate	140	2009, 2015 (S)	Insufficient	Mandatory (1996)
Denmark	145	2015 (S)	SAC	Adequate	101	2012 (S)	Insufficient	Mandatory (2000) ^f^
Finland	96	2017 (N)	Adults (25–74)	Insufficient	115	2013–2017 (S) ^f^	Insufficient	Voluntary ^f^
France	136	2006–2007 (N)	Adults (18–74)	Adequate	65	2006–2009 (S)	Insufficient	Voluntary
Germany	89	2014–2017 (N)	SAC, Adolescent (6–12)	Insufficient	54	2008–2011 (N) ^c^	Insufficient	Voluntary
Greece	132	2018 (N)	Adults	Adequate	127	2008–2015 (S)	Insufficient	Voluntary
Hungary	228	2005 (S)	SAC (10–14)	Adequate	128	2018 (S) ^d^	Insufficient	Mandatory (2013)
Ireland	111	2014–2015 (N)	Adolescent girls (14–15)	Adequate	107	2008–2010 (S)	Insufficient	Voluntary
Italy	118	2015–2019 (S)	SAC	Adequate	72	2002–2013 (S)	Insufficient	Mandatory (2005)
Netherlands	130	2006 (S)	Adults (50–72)	Adequate	223	2002–2006 (S)	Adequate	Voluntary
Poland	112	2009–2011 (S)	SAC (6–12)	Adequate	113	2007–2008 (S)	Insufficient	Mandatory (2010)
Portugal	106	2010 (N)	SAC	Adequate	85	2005–2007 (N)	Insufficient	Voluntary
Romania	255	2015–2016 (N)	SAC (6–11)	Adequate	206	2016 (S)	Adequate	Mandatory (2009)
Spain	173	2011–2012 (N)	SAC	Adequate	120	2002–2011 (S)	Insufficient	Voluntary
Sweden	125	2006–2007 (N)	SAC (6–12)	Adequate	98	2006–2007; 2010–2012 (S)	Insufficient	Voluntary (1936) ^f^
Switzerland	137	2015 (N)	SAC (6–12)	Adequate	136	2015 (N)	Insufficient	Voluntary
United Kingdom	166	2015–2016 (N)	SAC, Adolescent (4–18)	Adequate	99	2002–2011 (S)	Insufficient	no USI-program

Abbreviations: SAC, School-age children (typically 6–12 years old); UIC, urinary iodine concentration; USI, Universal salt iodization; N—Nationally-representative data; S—Sub-national data only; Dates according to ^a^ [56], ^b^ [42], ^c^ [57], ^d^ [43], ^e^ [49], ^f^ [50,51].

**Table 2:** Potential thyroid-disrupting chemicals (TDCs) targeting thyroid signaling pathway.

Examples of Chemicals	Target of TDCs Action and Results	Neurodevelopmental Alterations
Organochlorine pesticides (OCPs) ^1^ Polychlorinated biphenyl compounds (PCBs) ^2^	**TSH receptor signaling** and decreased stimulation of thyrocytes [105].	Impaired cognitive, motor and communication development [135,136,137,138,139] Impaired cognitive, motor development and playing activity [140] Reduced IQ [141] Development of ADHD-associated behavior [142]
Perchlorate ^3^ Thiocyanate ^3^ Nitrate ^3^ Phthalates ^4^	**Na+/I− symporter (NIS)** and inhibition of TH biosynthesis.	Impaired cognitive development [143] Pre- and postnatal exposures to tobacco may influence neurocognitive development [144] Sex-specific effects on cognitive, psychomotor, and behavioral development [145,146,147] Lower nonverbal and verbal IQ scores in offspring [148,149]
Propylthiouracil (PTU) Methimazole (MMI) Genistein ^5^ 4-Nonylphenol (NP) ^6^ Benzophenone 2 (BP2) ^7^ Pesticide (Amitrole) ^8^	**Inhibition of thyroid peroxidase (TPO)** results in decreased TH synthesis and subsequent reduction in circulating concentrations of THs.	Increased risk of periventricular heterotopia [150] TH Insufficiency Induces brain malformation and learning impairments [151] Decreased cognitive function [152]
OH-PCBs ^2^ Polybrominated diphenyl ethers (PBDEs) ^9^ Phthalates ^4^ Genistein ^5^	**TH-distributor proteins**: displacement of T4 and T3 by thyroid serum binding protein transthyretin (TTR) and/or thyroid binding globulin (TBG) disturbs the TH homeostasis and decreases plasma TH levels.	Impaired cognitive, behavioral, and motor development [153,154,155,156,157] Delayed neurodevelopment [158]
Polychlorinated biphenyls (PCBs, OH-PCBs) ^2^ Triclosan ^10^	Upregulation of **thyroid hormone catabolism** via the activation of hepatic nuclear receptors leads to a decrease in circulating TH levels [93,148].	Impaired early motor development [159] Hearing loss [160] Altered serum thyroid hormone levels [161,162]
Silymarin ^11^	Interference with cellular **transmembrane transporters** (MCT8, MCT10 and OATP1C1) inhibits T3 uptake.	Unwanted effects on the TH axis [163]
Erythrosine ^12^ 6 propylthiouracil PCBs ^2^	Modification of the **deiodinase enzyme activities** (DIO2, DIO3) by competitive inhibition of the enzyme or by interaction with its sulfhydryl cofactor.	With the exception of FD&C Red No. 3 dye, which causes thyroid tumors in rats, no studies to date have shown that chemicals that affect DIO expression and/or activity directly manifest in undesirable outcomes [105].

^1^ OCPs—especially used in agriculture to protect cultivated plants, but due to both their environmental persistence and neurotoxicity, their use has been banned or greatly reduced in the last decades. ^2^ PCBs—banned compounds used to make electrical equipment like transformers and in hydraulic fluids, heat transfer fluids, lubricants and plasticizers. ^3^ Perchlorate, thiocyanate, and nitrate—Individuals are exposed to these contaminants through food or other sources (e.g., cigarette smoke for thiocyanate or rocket propellant and fertilizers for perchlorate and nitrate). ^4^ Phthalates—used to make plastics more flexible, they are also found in some food packaging, cosmetics, children’s toys, and medical devices. ^5^ Genistein—a naturally occurring substance in plants with hormone-like activity found in soy products like tofu or soy milk. ^6^ 4NP—used in manufacturing antioxidants, lubricating oil additives, laundry and dish detergents, emulsifiers, and solubilizers. ^7^ BP2—is no longer permitted as a UV filter to be used in sun lotions within, for example, the European Union. However, it is still contained in plastic materials or many cosmetics to prevent UV-induced damage. ^8^ Amitrole—used as herbicide. ^9^ PBDEs—used to make flame retardants for household products such as furniture foam and carpets. Though most PBDEs have been banned or are being phased out, they are still persistent in the environment. ^10^ Triclosan—may be found in some anti-microbial and personal care products, like liquid body wash. ^11^ Silymarin—flavonoid mixture, a purified extract of the milk thistle. ^12^ Erythrosine, also known as Red No. 3, is an organoiodine compound. It is a pink dye that is primarily used for food coloring.

**Table 3:** Observational studies about adverse effects on cognitive development and behavioral disorders related to mild iodine deficiency—characteristics of all studies included in the systematic review [180]. (Sibling papers merged).

Author, Year [Reference]	Total Number of Participants Tested for Outcome	Country	Maternal Thyroid Dysfunction	Gestation at TFT	Criteria for Thyroid Dysfunction	Child Age at Assessment	Neurodevelopmental Outcome Measures
Pop et al., 1999 [68]	220	The Netherlands	HR	12 and 32 weeks	10th percentile fT4 (<10.4 pmol/L) and fifth percentile fT4 (<9.8 pmol/L)	10 months	Bayley Scales of Infant Development
Pop et al., 2003 [181]	125	The Netherlands	HR	12, 24 and 32 weeks	fT4 <10th Percentile (12.10 pmol/L)	1–2 years	Bayley Scale of Infant Development
Kasatkina et al., 2006 [95]	35	Russia	HR	1st and 3rd trimesters	fT4 < 12.0 pmol/L	6, 9 and 12 months	Gnome method, in particular, the Coefficient of Mental Development
Li et al., 2010 [182]	213	China	SH and HR	16 to 20 weeks	SH = TSH > 97.50th percentile (4.21 mU/L), HR = tT4 < 2.50th percentile (101.79 nmol/L)	25–30 months	Bayley Scale of Infant Development
Henrichs et al., 2010 [183]	3659	The Netherlands	HR and Co TSH	13.3 weeks	HR = fT4 10th percentile (<11.76 pmol/L) and fifth percentile (<10.96 pmol/L), Co TSH = TSH ref range 0.03–2.50 mU/L	18 and 30 months	MacArthur Communicative Development Inventory at 18 months, Language Development Survey at 30 months
Suárez-Rodríguez et al., 2012 [94]	70	Spain	HR	37 weeks	fT4 < 10th percentile (9.5 pmol/L)	38 months and 5 years	The McCarthy Scales of Children’s Abilities
Williams et al., 2012 [184]	166	UK	SH and HR	±1 h after birth	SH = TSH > 3.0 mU/L, HR = fT4 ≤ 10th percentile (11.6 pmol/L) or tT4 ≤10th percentile (108.4 nmol/L)	5.5 years	The McCarthy Scales of Children’s Abilities
Craig et al., 2012 [185]	196	USA	HR	2nd trimester	fT4 < 3rd Percentile (11.84 pmol/L)	2 years	Bayley’s Scale of Infant Development III
Ghassabian et al., 2014 [93]/Korevaar et al., 2016 [96]	3737/5647	The Netherlands	HR and SH	13.5/13.2 weeks	HR = fT4 < 5th percentile (10.99 pmol/L), SH= TSH > 2.50 mU/L	6 years	Snijders-Oomen Niet-verbale intelligence test, revisie (Mosaics and Categories)
Päkkilä et al., 2015 [186]	5295	Finland	HR, SH and OH	Mean 10.7 weeks	HR = fT4 < 11.4–11.09 pmol/L depending upon trimester, SH = TSH > 3.10–3.50 mU/L depending upon trimester	8 and 16 years	Strengths and Weaknesses of ADHD Symptoms and Normal Behavior, Teacher reported child school performance, Youth Self Report and WISC-Revised
Grau et al., 2015 [187]	455	Spain	HR	1st and 2nd trimesters	<10th Percentile (13.7–11.5 pmol/L depending on trimester)	1 and 6–8 years	Brunet-Lezine scale and WISC-IV

Abbreviations: HR = Hypothyroxinemia, OH = Overt hypothyroidism, SH = Subclinical hypothyroidism, TFT = Thyroid function tests, Co = Continuous, TSH = Thyroid stimulating hormone.

## Data Availability

Not applicable.

## References

[1] Eastman C.J., Zimmermann M.B., Feingold K.R., Anawalt B., Boyce A., Chrousos G., de Herder W.W., Dhatariya K., Dungan K., Hershman J.M., Hofland J., Kalra S. (2000). The iodine deficiency disorders. Endotext [Internet].

[2] American College of Obstetricians and Gynecologists’ Committee on Obstetric Practice (2021). Reducing Prenatal Exposure to Toxic Environmental Agents: ACOG Committee Opinion, Number 832. Obstet. Gynecol..

[3] Yilmaz B., Terekeci H., Sandal S., Kelestimur F. (2020). Endocrine disrupting chemicals: Exposure, effects on human health, mechanism of action, models for testing and strategies for prevention. Rev. Endocr. Metab. Disord..

[4] Ghassabian A., Trasande L. (2018). Disruption in Thyroid Signaling Pathway: A Mechanism for the Effect of Endocrine-Disrupting Chemicals on Child Neurodevelopment. Front. Endocrinol..

[5] Gómez-Roig M.D., Pascal R., Cahuana M.J., García-Algar O., Sebastiani G., Andreu-Fernández V., Martínez L., Rodríguez G., Iglesia I., Ortiz-Arrabal O. (2021). Environmental Exposure during Pregnancy: Influence on Prenatal Development and Early Life: A Comprehensive Review. Fetal Diagn. Ther..

[6] Köhrle J., Frädrich C. (2021). Thyroid hormone system disrupting chemicals. Best Pract. Res. Clin. Endocrinol. Metab..

[7] Hamers T., Kortenkamp A., Scholze M., Molenaar D., Cenijn P.H., Weiss J.M. (2020). Transthyretin-Binding Activity of Complex Mixtures Representing the Composition of Thyroid-Hormone Disrupting Contaminants in House Dust and Human Serum. Environ. Health Perspect..

[8] Lisco G., De Tullio A., Giagulli V.A., De Pergola G., Triggiani V. (2020). Interference on iodine uptake and human thyroid function by perchlorate-contaminated water and food. Nutrients.

[9] Demeneix B.A. (2019). Evidence for Prenatal Exposure to Thyroid Disruptors and Adverse Effects on Brain Development. Eur. Thyroid J..

[10] Ghassabian A., Pierotti L., Basterrechea M., Chatzi L., Estarlich M., Fernández-Somoano A., Fleisch A.F., Gold D.R., Julvez J., Karakosta P. (2019). Association of Exposure to Ambient Air Pollution with Thyroid Function During Pregnancy. JAMA Netw. Open.

[11] Mughal B.B., Fini J.B., Demeneix B.A. (2018). Thyroid-disrupting chemicals and brain development: An update. Endocr. Connect..

[12] Lanphear B.P. (2015). The impact of toxins on the developing brain. Annu. Rev. Public Health.

[13] Hetzel B.S. (2012). The development of a global program for the elimination of brain damage due to iodine deficiency. Asia Pac. J. Clin. Nutr..

[14] Monahan M., Boelaert K., Jolly K., Chan S., Barton P., Roberts T.E. (2015). Costs and benefits of iodine supplementation for pregnant women in a mildly to moderately iodine-deficient population: A modelling analysis. Lancet Diabetes Endocrinol..

[15] Großklaus R. (2007). Nutzen und Risiken der Jodprophylaxe. Einfluss von Jodsalz auf Schilddrüsenkrankheiten und die Gesundheit des Menschen. Prävent. Gesundh..

[16] Demeneix B., Slama R. (2019). Endocrine Disruptors: From Scientific Evidence to Human Health Protection. Report Commissioned by the PETI Committee of the European Parliament.

[17] Trasande L., Zoeller R.T., Hass U., Kortenkamp A., Grandjean P., Myers J.P., DiGangi J., Hunt P.M., Rudel R., Sathyanarayana S. (2016). Burden of disease and costs of exposure to endocrine disrupting chemicals in the European Union: An updated analysis. Andrology.

[18] Costa L.G., Cole T.B., Dao K., Chang Y.-C., Garrick J.M. (2019). Developmental impact of air pollution on brain function. Neurochem. Int..

[19] Rivas I., Basagaña X., Cirach M., López-Vicente M., Suades-González E., Garcia-Esteban R., Álvarez-Pedrerol M., Dadvand P., Sunyer J. (2019). Association between Early Life Exposure to Air Pollution and Working Memory and Attention. Environ. Health Perspect..

[20] D’Angiulli A. (2018). Severe Urban Outdoor Air Pollution and Children’s Structural and Functional Brain Development, From Evidence to Precautionary Strategic Action. Front. Public Health.

[21] Suades-González E., Gascon M., Guxens M., Sunyer J. (2015). Air Pollution and Neuropsychological Development: A Review of the Latest Evidence. Endocrinology.

[22] Padmanabhan V., Song W., Puttabyatappa M. (2021). Pregnatio Perturbatio-Impact of Endocrine-Disrupting Chemicals. Endocr. Rev..

[23] Inoue K., Yan Q., Arah O.A., Paul K., Walker D.I., Jones D.P., Ritz B. (2020). Air Pollution and Adverse Pregnancy and Birth Outcomes: Mediation Analysis Using Metabolomic Profiles. Curr. Environ. Health Rep..

[24] Pergialiotis V., Kotrogianni P., Christopoulos-Timogiannakis E., Koutaki D., Daskalakis G., Papantoniou N. (2018). Bisphenol A and adverse pregnancy outcomes: A systematic review of the literature. J. Matern. Fetal Neonatal Med..

[25] Janssen B.G., Saenen N.D., Roels H.A., Madhloum N., Gyselaers W., Lefebvre W., Penders J., Vanpoucke C., Vrijens K., Nawrot T.S. (2017). Fetal Thyroid Function, Birth Weight, and in Utero Exposure to Fine Particle Air Pollution: A Birth Cohort Study. Environ. Health Perspect..

[26] Zhao X., Peng S., Xiang Y., Yang Y., Li J., Shan Z., Teng W. (2017). Correlation between Prenatal Exposure to Polybrominated Diphenyl Ethers (PBDEs) and Infant Birth Outcomes: A Meta-Analysis and an Experimental Study. Int. J. Environ. Res. Public Health.

[27] Li M., Eastman C.J. (2012). The changing epidemiology of iodine deficiency. Nat. Rev. Endocrinol..

[28] Velasco I., Bath S.C., Rayman M.P. (2018). Iodine as Essential Nutrient during the First 1000 Days of Life. Nutrients.

[29] Bath S.C. (2019). The effect of iodine deficiency during pregnancy on child development. Proc. Nutr. Soc..

[30] Min H., Dong J., Wang Y., Wang Y., Teng W., Xi Q., Chen J. (2016). Maternal Hypothyroxinemia-Induced Neurodevelopmental Impairments in the Progeny. Mol. Neurobiol..

[31] De Escobar G.M., Obregón M.J., del Rey F.E. (2007). Iodine deficiency and brain development in the first half of pregnancy. Public Health Nutr..

[32] Chen Y., Xue F. (2020). The impact of gestational hypothyroxinemia on the cognitive and motor development of offspring. J. Matern. Fetal Neonatal Med..

[33] Dosiou C., Medici M. (2017). Isolated maternal hypothyroxinemia during pregnancy: Knowns and unknowns. Eur. J. Endocrinol..

[34] Henrichs J., Ghassabian A., Peeters R.P., Tiemeier H. (2013). Maternal hypothyroxinemia and effects on cognitive functioning in childhood: How and why?. Clin. Endocrinol..

[35] Moleti M., Trimarchi F., Vermiglio F. (2011). Doubts and Concerns about Isolated Maternal Hypothyroxinemia. J. Thyroid Res..

[36] Munn Z., Peters M.D.J., Stern C., Tufanaru C., McArthur A., Aromataris E. (2018). Systematic review or scoping review? Guidance for authors when choosing between a systematic or scoping review approach. BMC Med. Res. Methodol..

[37] Glinoer D. (2004). The regulation of thyroid function during normal pregnancy: Importance of the iodine nutrition status. Best Pract. Res. Clin. Endocrinol. Metab..

[38] EFSA NDA Panel (EFSA Panel on Panel on Dietetic Products Nutrition and Allergies) (2014). Scientific Opinion on Dietary Reference Values for iodine. EFSA J..

[39] World Health Organization (2007). Assessment of Iodine Deficiency Disorders and Monitoring Their Elimination: A Guide for Programme Managers.

[40] Eastman C.J., Ma G., Li M. (2019). Optimal Assessment and Quantification of Iodine Nutrition in Pregnancy and Lactation: Laboratory and Clinical Methods, Controversies and Future Directions. Nutrients.

[41] Gizak M., Rogers L., Gorstein J.A., Zimmermann M., Andersson M. Global iodine status in school-age children, women of reproductive age, and pregnant women in 2017. Proceedings of the Poster Presented at the Nutrition 2018, the American Society for Nutrition Annual Conference.

[42] The Iodine Global Network (2017). Global Scorecard of Iodine Nutrition in 2017 in the General Population and in Pregnant Women (PW).

[43] Katko M., Gazso A.A., Hircsu I., Bhattoa H.P., Molnar Z., Kovacs B., Andrasi D., Aranyosi J., Makai R., Veress L. (2018). Thyroglobulin level at week 16 of pregnancy is superior to urinary iodine concentration in revealing preconceptual and first trimester iodine supply. Matern. Child Nutr..

[44] Candido A.C., Morais N.D.S.D., Dutra L.V., Pinto C.A., Franceschini S.D.C.C., Alfenas R.D.C.G. (2019). Insufficient iodine intake in pregnant women in different regions of the world: A systematic review. Arch. Endocrinol. Metab..

[45] Baldini E., Virili C., D’Armiento E., Centanni M., Ulisse S. (2019). Iodine Status in Schoolchildren and Pregnant Women of Lazio, a Central Region of Italy. Nutrients.

[46] Medici M., Ghassabian A., Visser W.E., Keizer-Schrama S.M.P.F.D.M., Jaddoe V.W.V., Hooijkaas H., Hofman A., Steegers E.A.P., Bongers-Schokking J.J., Ross H.A. (2014). Women with high early pregnancy urinary iodine levels have an increased risk of hyperthyroid newborns: The population-based Generation R Study. Clin. Endocrinol..

[47] Vandevijvere S., Amsalkhir S., Mourri A.B., Van Oyen H., Moreno-Reyes R. (2013). Iodine deficiency among Belgian pregnant women not fully corrected by iodine-containing multivitamins: A national cross-sectional survey. Br. J. Nutr..

[48] Andersen S.L., Sørensen L.K., Krejbjerg A., Møller M., Laurberg P. (2013). Iodine deficiency in Danish pregnant women. Dan. Med. J..

[49] Global Fortification Data Exchange. https://fortificationdata.org.

[50] Nyström H.F., Brantsæter A.L., Erlund I., Gunnarsdottir I., Hulthén L., Laurberg P., Mattisson I., Rasmussen L.B., Virtanen S., Meltzer H.M. (2016). Iodine status in the Nordic countries—Past and present. Food Nutr. Res..

[51] Miles E.A., Vahlberg T., Calder P.C., Houttu N., Pajunen L., Koivuniemi E., Mokkala K., Laitinen K. (2022). Iodine status in pregnant women and infants in Finland. Eur. J. Nutr..

[52] Nazeri P., Mirmiran P., Shiva N., Mehrabi Y., Mojarrad M., Azizi F. (2015). Iodine nutrition status in lactating mothers residing in countries with mandatory and voluntary iodine fortification programs: An updated systematic review. Thyroid.

[53] Manousou S., Andersson M., Eggertsen R., Hunziker S., Hulthén L., Nyström H.F. (2020). Iodine deficiency in pregnant women in Sweden: A national cross-sectional study. Eur. J. Nutr..

[54] Lindorfer H., Krebs M., Kautzky-Willer A.A., Bancher-Todesca D., Sager M., Gessl A.A. (2015). Iodine deficiency in pregnant women in Austria. Eur. J. Clin. Nutr..

[55] The Iodine Global Network (2020). Global Scorecard of Iodine Nutrition in 2020 in the General Population Based on School-Age Children (SAC).

[56] Johner S.A., Thamm M., Schmitz R., Remer T. (2016). Examination of iodine status in the German population: An example for methodological pitfalls of the current approach of iodine status assessment. Eur. J. Nutr..

[57] Ittermann T., Albrecht D., Arohonka P., Bilek R., Castro J.J., Dahl L., Nystrom H.F., Gaberscek S., Garcia-Fuentes E., Gheorghiu M.L. (2020). Standardized Map of Iodine Status in Europe. Thyroid.

[58] Hey I., Thamm M. (2019). Monitoring der Jod- und Natriumversorgung bei Kindern und Jugendlichen im Rahmen der Studie des Robert Koch-Instituts zur Gesundheit von Kindern und Jugendlichen in Deutschland (KiGGS Welle 2).

[59] Bissinger K., Herrmann R., Jordan I. (2022). Salt iodisation of processed foods in Germany: Evidence, processors’ perceptions and implications for public health. Br. Food J..

[60] Olivieri A., Di Cosmo C., De Angelis S., Da Cas R., Stacchini P., Pastorelli A., Vitti P., Prevention R.O.F.G. (2017). Regional Observatories for Goiter Prevention. The way forward in Italy for iodine. Minerva Med..

[61] Szybinski Z. Poland Remains Iodine Sufficient after 20 Years of IDD Prevention, but Pregnant Women May Be at Risk. IDD Newsletter, 1 August 2015. https://ign.org/research/.

[62] Gerasimov G. Progress of IDD Elimination through Universal Salt Iodization in the Czech Republic, Slovakia, Hungary and Poland. UNICEF Regional Office for Central and Eastern Europe, Commonwealth of Independent States and the Baltic States, March 2002. https://www.ign.org/cm_data/2002_Gerasimov_CWCP_Salt_IDDreport2002.pdf.

[63] Kirkegaard-Klitbo D.M., Perslev K., Andersen S.L., Perrild H., Knudsen N., Weber T., Rasmussen L.B., Laurberg P. (2016). Iodine deficiency in pregnancy is prevalent in vulnerable groups in Denmark. Dan. Med. J..

[64] Vandevijvere S., Mourri A.B., Amsalkhir S., Avni F., Van Oyen H., Moreno-Reyes R. (2012). Fortification of bread with iodized salt corrected iodine deficiency in school-aged children, but not in their mothers: A national cross-sectional survey in Belgium. Thyroid.

[65] Trumpff C., De Schepper J., Tafforeau J., Van Oyen H., Vanderfaeillie J., Vandevijvere S. (2013). Mild iodine deficiency in pregnancy in Europe and its consequences for cognitive and psychomotor development of children: A review. J. Trace Elem. Med. Biol..

[66] Zimmermann M., Delange F. (2004). Iodine supplementation of pregnant women in Europe: A review and recommendations. Eur. J. Clin. Nutr..

[67] Panth P., Guerin G., DiMarco N.M. (2019). A Review of Iodine Status of Women of Reproductive Age in the USA. Biol. Trace Elem. Res..

[68] Pop V.J., Kuijpens J.L., van Baar A., Verkerk G., Van Son M.M., De Vijlder J.J., Vulsma T., Wiersinga W.M., Drexhage H.A., Vader H.L. (1999). Low maternal free thyroxine concentrations during early pregnancy are associated with impaired psychomotor development in infancy. Clin. Endocrinol..

[69] Alexander E.K., Pearce E.N., Brent G.A., Brown R.S., Chen H., Dosiou C., Grobman W.A., Laurberg P., Lazarus J.H., Mandel S.J. (2017). 2017 Guidelines of the American Thyroid Association for the Diagnosis and Management of Thyroid Disease During Pregnancy and the Postpartum. Thyroid.

[70] Furnica R.M., Lazarus J.H., Gruson D., Daumerie C. (2015). Update on a new controversy in endocrinology: Isolated maternal hypothyroxinemia. J. Endocrinol. Investig..

[71] Dong A.C., Stagnaro-Green A. (2019). Differences in Diagnostic Criteria Mask the True Prevalence of Thyroid Disease in Pregnancy: A Systematic Review and Meta-Analysis. Thyroid.

[72] Elahi S., Nagra S.A. (2014). Low maternal iodine intake and early pregnancy hypothyroxinemia: Possible repercussions for children. Indian J. Endocrinol. Metab..

[73] Moog N., Entringer S., Heim C., Wadhwa P., Kathmann N., Buss C. (2017). Influence of maternal thyroid hormones during gestation on fetal brain development. Neuroscience.

[74] Gilbert M.E., O’Shaughnessy K.L., Axelstad M. (2020). Regulation of Thyroid-disrupting Chemicals to Protect the Developing Brain. Endocrinology.

[75] López-Muñoz E., Mateos-Sánchez L., Mejía-Terrazas G.E., Bedwell-Cordero S.E. (2019). Hypothyroidism and isolated hypothyroxinemia in pregnancy, from physiology to the clinic. Taiwan J. Obstet. Gynecol..

[76] Korevaar T.I., Nieboer D., Bisschop P.H., Goddijn M., Medici M., Chaker L., de Rijke Y.B., Jaddoe V.W., Visser T.J., Steyerberg E.W. (2016). Risk factors and a clinical prediction model for low maternal thyroid function during early pregnancy: Two population-based prospective cohort studies. Clin. Endocrinol..

[77] Liu Y., Li G., Guo N., Liu X., Huang S., Du Q. (2022). Association between Maternal Characteristics and the Risk of Isolated Maternal Hypothyroxinemia. Front. Endocrinol..

[78] Karbownik-Lewińska M., Stępniak J., Lewiński A. (2021). Potential Risk Factors for Isolated Hypothyroxinemia in Women of Childbearing Age-Results from Retrospective Analysis. J. Clin. Med..

[79] Etemadi A., Amouzegar A., Mehran L., Tohidi M., Azizi F., Moradi K., Delshad H. (2020). Isolated Hypothyroxinemia in Iranian Pregnant Women, the Role of Iodine Deficiency: A Population-Based Cross-Sectional Study. Thyroid.

[80] Knight B.A., Shields B.M., Hattersley A.T., Vaidya B. (2016). Maternal hypothyroxinaemia in pregnancy is associated with obesity and adverse maternal metabolic parameters. Eur. J. Endocrinol..

[81] Ventura M., Melo M., Carrilho F. (2017). Selenium and Thyroid Disease: From Pathophysiology to Treatment. Int. J. Endocrinol..

[82] Teng X., Shan Z., Li C., Yu X., Mao J., Wang W., Xie X., Du J., Zhang S., Gao Z. (2018). Iron Deficiency May Predict Greater Risk for Hypothyroxinemia: A Retrospective Cohort Study of Pregnant Women in China. Thyroid.

[83] Hu X., Wang R., Shan Z., Dong Y., Zheng H., Jesse F.F., Rao E., Takahashi E., Li W., Teng W. (2016). Perinatal Iron Deficiency-Induced Hypothyroxinemia Impairs Early Brain Development Regardless of Normal Iron Levels in the Neonatal Brain. Thyroid.

[84] Soldin O.P., Soldin S.J. (2011). Thyroid hormone testing by tandem mass spectrometry. Clin. Biochem..

[85] Lazarus J., Brown R.S., Daumerie C., Hubalewska-Dydejczyk A., Negro R., Vaidya B. (2014). 2014 European thyroid association guidelines for the management of subclinical hypothyroidism in pregnancy and in children. Eur. Thyroid J..

[86] Miranda A., Sousa N. (2018). Maternal hormonal milieu influence on fetal brain development. Brain Behav..

[87] Bernal J., Feingold K.R., Anawalt B., Boyce A., Chrousos G., de Herder W.W., Dhatariya K., Dungan K., Hershman J.M., Hofland J., Kalra S. (2022). Thyroid Hormones in Brain Development and Function. Endotext [Internet].

[88] Korevaar T.I.M., Medici M., Visser T.J., Peeters R.P. (2017). Thyroid disease in pregnancy: New insights in diagnosis and clinical management. Nat. Rev. Endocrinol..

[89] Williams G.R. (2008). Neurodevelopmental and neurophysiological actions of thyroid hormone. J. Neuroendocrinol..

[90] Stepien B.K., Huttner W.B. (2019). Transport, Metabolism, and Function of Thyroid Hormones in the Developing Mammalian Brain. Front. Endocrinol..

[91] Costeira M.J., Oliveira P., Santos N.C., Ares S., Saenz-Rico B., de Escobar G.M., Palha J.A. (2011). Psychomotor development of children from an iodine-deficient region. J. Pediatr..

[92] Kawahori K., Hashimoto K., Yuan X., Tsujimoto K., Hanzawa N., Hamaguchi M., Kase S., Fujita K., Tagawa K., Okazawa H. (2018). Mild Maternal Hypothyroxinemia During Pregnancy Induces Persistent DNA Hypermethylation in the Hippocampal Brain-Derived Neurotrophic Factor Gene in Mouse Offspring. Thyroid.

[93] Ghassabian A., El Marroun H., Peeters R.P., Jaddoe V.W., Hofman A., Verhulst F.C., Tiemeier H., White T. (2014). Downstream effects of maternal hypothyroxinemia in early pregnancy: Nonverbal IQ and brain morphology in school-age children. J. Clin. Endocrinol. Metab..

[94] Suárez-Rodríguez M., Azcona-San Julián C., Alzina de Aguilar V. (2012). Hypothyroxinemia during pregnancy: The effect on neurodevelopment in the child. Int. J. Dev. Neurosci..

[95] Kasatkina E.P., Samsonova L.N., Ivakhnenko V.N., Ibragimova G.V., Ryabykh A.V., Naumenko L.L., Evdokimova Y.A. (2006). Gestational hypothyroxinemia and cognitive function in offspring. Neurosci. Behav. Physiol..

[96] Korevaar T.I.M., Muetzel R., Medici M., Chaker L., Jaddoe V.W.V., de Rijke Y.B., Steegers E.A.P., Visser T.J., White T., Tiemeier H. (2016). Association of maternal thyroid function during early pregnancy with offspring IQ and brain morphology in childhood: A population-based prospective cohort study. Lancet Diabetes Endocrinol..

[97] Lischinsky J.E., Skocic J., Clairman H., Rovet J. (2016). Preliminary Findings Show Maternal Hypothyroidism May Contribute to Abnormal Cortical Morphology in Offspring. Front. Endocrinol..

[98] Jansen T.A., Korevaar T.I.M., Mulder T.A., White T., Muetzel R.L., Peeters R.P., Tiemeier H. (2019). Maternal thyroid function during pregnancy and child brain morphology: A time window-specific analysis of a prospective cohort. Lancet Diabetes Endocrinol..

[99] Andersen S.L., Carlé A., Karmisholt J., Pedersen I.B., Andersen S. (2017). Mechanism in endocrinolgy: Neurodevelopmental disorders in children born to mothers with thyroid dysfunction: Evidence of fetal programming?. Eur. J. Endocrinol..

[100] Marty S., Beekhuijzen M., Charlton A., Hallmark N., Hannas B.R., Jacobi S., Melching-Kollmuss S., Sauer U.G., Sheets L.P., Strauss V. (2021). Towards a science-based testing strategy to identify maternal thyroid hormone imbalance and neurodevelopmental effects in the progeny—Part II: How can key events of relevant adverse outcome pathways be addressed in toxicological assessments?. Crit. Rev. Toxicol..

[101] Mulder T.A., Korevaar T.I., Peeters R.P., van Herwaarden A.E., de Rijke Y.B., White T., Tiemeier H. (2021). Urinary Iodine Concentrations in Pregnant Women and Offspring Brain Morphology. Thyroid.

[102] Boas M., Feldt-Rasmussen U., Main K.M. (2012). Thyroid effects of endocrine disrupting chemicals. Mol. Cell. Endocrinol..

[103] Jugan M.L., Levi Y., Blondeau J.P. (2010). Endocrine disruptors and thyroid hormone physiology. Biochem. Pharmacol..

[104] Hartoft-Nielsen M.-L., Boas M., Bliddal S., Rasmussen K., Main K., Feldt-Rasmussen U. (2011). Do Thyroid Disrupting Chemicals Influence Foetal Development during Pregnancy?. J. Thyroid Res..

[105] Noyes P.D., Friedman K.P., Browne P., Haselman J.T., Gilbert M.E., Hornung M.W., Barone S., Crofton K.M., Laws S.C., Stoker T.E. (2019). Evaluating Chemicals for Thyroid Disruption: Opportunities and Challenges with in Vitro Testing and Adverse Outcome Pathway Approaches. Environ. Health Perspect..

[106] Calsolaro V., Pasqualetti G., Niccolai F., Caraccio N., Monzani F. (2017). Thyroid Disrupting Chemicals. Int. J. Mol. Sci..

[107] Duntas L.H., Stathatos N. (2015). Toxic chemicals and thyroid function: Hard facts and lateral thinking. Rev. Endocr. Metab. Disord..

[108] Gilbert M.E., Rovet J., Chen Z., Koibuchi N. (2012). Developmental thyroid hormone disruption: Prevalence, environmental contaminants and neurodevelopmental consequences. Neurotoxicology.

[109] Heindel J.J., Balbus J., Birnbaum L., Brune-Drisse M.N., Grandjean P., Gray K., Landrigan P.J., Sly P.D., Suk W.A., Cory Slechta D. (2015). Developmental Origins of Health and Disease: Integrating Environmental Influences. Endocrinology.

[110] Neven K.Y., Cox B., Cosemans C., Gyselaers W., Penders J., Plusquin M., Roels H.A., Vrijens K., Ruttens A., Nawrot T.S. (2021). Lower iodine storage in the placenta is associated with gestational diabetes mellitus. BMC Med..

[111] Neven K.Y., Wang C., Janssen B.G., Roels H.A., Vanpoucke C., Ruttens A., Nawrot T.S. (2021). Ambient air pollution exposure during the late gestational period is linked with lower placental iodine load in a Belgian birth cohort. Environ. Int..

[112] Neven K.Y., Cox B., Vrijens K., Plusquin M., Roels H.A., Ruttens A., Nawrot T.S. (2020). Determinants of placental iodine concentrations in a mild-to-moderate iodine-deficient population: An ENVIRONAGE cohort study. J. Transl. Med..

[113] Di N., He W., Zhang K., Cui J., Chen J., Cheng J., Chu B., Li S., Xie Y., Xiang H. (2021). Association of short-term air pollution with systemic inflammatory biomarkers in routine blood test: A longitudinal study. Environ. Res. Lett..

[114] Kelley A.S., Banker M., Goodrich J.M., Dolinoy D.C., Burant C., Domino S.E., Smith Y.R., Song P.X.K., Padmanabhan V. (2019). Early pregnancy exposure to endocrine disrupting chemical mixtures are associated with inflammatory changes in maternal and neonatal circulation. Sci. Rep..

[115] Saenen N.D., Martens D.S., Neven K.Y., Alfano R., Bové H., Janssen B.G., Roels H.A., Plusquin M., Vrijens K., Nawrot T.S. (2019). Air pollution-induced placental alterations: An interplay of oxidative stress, epigenetics, and the aging phenotype?. Clin. Epigenet..

[116] Yan Q., Liew Z., Uppal K., Cui X., Ling C., Heck J.E., von Ehrenstein O.S., Wu J., Walker D.I., Jones D.P. (2019). Maternal serum metabolome and traffic-related air pollution exposure in pregnancy. Environ. Int..

[117] Zota A.R., Geller R.J., Romano L.E., Coleman-Phox K., Adler N.E., Parry E., Wang M., Park J.-S., Elmi A.F., Laraia B.A. (2018). Association between persistent endocrine-disrupting chemicals (PBDEs, OH-PBDEs, PCBs, and PFASs) and biomarkers of inflammation and cellular aging during pregnancy and postpartum. Environ. Int..

[118] Holland N., Huen K., Tran V., Street K., Nguyen B., Bradman A., Eskenazi B. (2016). Urinary Phthalate Metabolites and Biomarkers of Oxidative Stress in a Mexican-American Cohort: Variability in Early and Late Pregnancy. Toxics.

[119] Ferguson K.K., Cantonwine D.E., McElrath T.F., Mukherjee B., Meeker J.D. (2016). Repeated measures analysis of associations between urinary bisphenol-A concentrations and biomarkers of inflammation and oxidative stress in pregnancy. Reprod. Toxicol..

[120] Zhou M., Ford B., Lee D., Tindula G., Huen K., Tran V., Bradman A., Gunier R., Eskenazi B., Nomura D.K. (2018). Metabolomic Markers of Phthalate Exposure in Plasma and Urine of Pregnant Women. Front. Public Health.

[121] Chen H., Oliver B.G., Pant A., Olivera A., Poronnik P., Pollock C.A., Saad S. (2021). Particulate Matter, an Intrauterine Toxin Affecting Foetal Development and Beyond. Antioxidants.

[122] Johnson N.M., Hoffmann A.R., Behlen J.C., Lau C., Pendleton D., Harvey N., Shore R., Li Y., Chen J., Tian Y. (2021). Air pollution and children’s health-a review of adverse effects associated with prenatal exposure from fine to ultrafine particulate matter. Environ. Health Prev. Med..

[123] Costa L.G., Cole T.B., Dao K., Chang Y.-C., Coburn J., Garrick J.M. (2020). Effects of air pollution on the nervous system and its possible role in neurodevelopmental and neurodegenerative disorders. Pharmacol. Ther..

[124] Ozaki K., Kato D., Ikegami A., Hashimoto A., Sugio S., Guo Z., Shibushita M., Tatematsu T., Haruwaka K., Moorhouse A.J. (2020). Maternal immune activation induces sustained changes in fetal microglia motility. Sci. Rep..

[125] Street M.E., Bernasconi S. (2020). Endocrine-Disrupting Chemicals in Human Fetal Growth. Int. J. Mol. Sci..

[126] Genc S., Zadeoglulari Z., Fuss S.H., Genc K. (2012). The adverse effects of air pollution on the nervous system. J. Toxicol..

[127] Fini J.-B., Mughal B.B., Le Mével S., Leemans M., Lettmann M., Spirhanzlova P., Affaticati P., Jenett A., Demeneix B.A. (2017). Human amniotic fluid contaminants alter thyroid hormone signalling and early brain development in Xenopus embryos. Sci. Rep..

[128] Mitro S.D., Johnson T., Zota A.R. (2015). Cumulative Chemical Exposures During Pregnancy and Early Development. Curr. Environ. Health Rep..

[129] De Renzy-Martin K.T., Frederiksen H., Christensen J.S., Kyhl H.B., Andersson A.-M., Husby S., Barington T., Main K.M., Jensen T.K. (2014). Current exposure of 200 pregnant Danish women to phthalates, parabens and phenols. Reproduction.

[130] Bose S., Ross K.R., Rosa M.J., Chiu Y.-H.M., Just A., Kloog I., Wilson A., Thompson J., Svensson K., Rojo M.M.T. (2019). Prenatal particulate air pollution exposure and sleep disruption in preschoolers: Windows of susceptibility. Environ. Int..

[131] Vandenberg L.N., Colborn T., Hayes T.B., Heindel J.J., Jacobs D.R., Lee D.-H., Shioda T., Soto A.M., vom Saal F.S., Welshons W.V. (2012). Hormones and endocrine-disrupting chemicals: Low-dose effects and nonmonotonic dose responses. Endocr. Rev..

[132] Dutta S., Haggerty D.K., Rappolee D.A., Ruden D.M. (2020). Phthalate Exposure and Long-Term Epigenomic Consequences: A Review. Front. Genet..

[133] Préau L., Fini J.-B., Morvan-Dubois G., Demeneix B. (2015). Thyroid hormone signaling during early neurogenesis and its significance as a vulnerable window for endocrine disruption. Biochim. Biophys. Acta.

[134] Xin F., Susiarjo M., Bartolomei M.S. (2015). Multigenerational and transgenerational effects of endocrine disrupting chemicals: A role for altered epigenetic regulation?. Semin. Cell Dev. Biol..

[135] Bouchard M.F., Chevrier J., Harley K.G., Kogut K., Vedar M., Calderon N., Trujillo C., Johnson C., Bradman A., Barr D.B. (2011). Prenatal exposure to organophosphate pesticides and IQ in 7-year-old children. Environ. Health Perspect..

[136] Eskenazi B., Kogut K., Huen K., Harley K.G., Bouchard M., Bradman A., Boyd-Barr D., Johnson C., Holland N. (2014). Organophosphate pesticide exposure, PON1, and neurodevelopment in school-age children from the CHAMACOS study. Environ. Res..

[137] Yamazaki K., Araki A., Nakajima S., Miyashita C., Ikeno T., Itoh S., Minatoya M., Kobayashi S., Mizutani F., Chisaki Y. (2018). Association between prenatal exposure to organochlorine pesticides and the mental and psychomotor development of infants at ages 6 and 18 months: The Hokkaido Study on Environment and Children’s Health. Neurotoxicology.

[138] Wang S., Hu C., Lu A., Wang Y., Cao L., Wu W., Li H., Wu M., Yan C. (2021). Association between prenatal exposure to persistent organic pollutants and neurodevelopment in early life: A mother-child cohort (Shanghai, China). Ecotoxicol. Environ. Saf..

[139] Jeddy Z., Kordas K., Allen K., Taylor E.V., Northstone K., Flanders W.D., Namulanda G., Sjodin A., Hartman T.J. (2018). Prenatal exposure to organochlorine pesticides and early childhood communication development in British girls. Neurotoxicology.

[140] Vermeir G., Covaci A., Van Larebeke N., Schoeters G., Nelen V., Koppen G., Viaene M. (2021). Neurobehavioural and cognitive effects of prenatal exposure to organochlorine compounds in three year old children. BMC Pediatr..

[141] Jacobson J.L., Jacobson S.W. (1996). Intellectual impairment in children exposed to polychlorinated biphenyls in utero. N. Engl. J. Med..

[142] Sagiv S.K., Thurston S.W., Bellinger D.C., Tolbert P.E., Altshul L.M., Korrick S.A. (2010). Prenatal organochlorine exposure and behaviors associated with attention deficit hyperactivity disorder in school-aged children. Am. J. Epidemiol..

[143] Taylor P., Okosieme O.E., Murphy R., Hales C., Chiusano E., Maina A., Joomun M., Bestwick J.P., Smyth P., Paradice R. (2014). Maternal perchlorate levels in women with borderline thyroid function during pregnancy and the cognitive development of their offspring: Data from the Controlled Antenatal Thyroid Study. J. Clin. Endocrinol. Metab..

[144] Moore B.F., Shapiro A.L., Wilkening G., Magzamen S., Starling A.P., Allshouse W.B., Adgate J.L., Dabelea D. (2020). Prenatal Exposure to Tobacco and Offspring Neurocognitive Development in the Healthy Start Study. J. Pediatr..

[145] Daniel S., Balalian A.A., Insel B.J., Liu X., Whyatt R.M., Calafat A.M., Rauh V.A., Perera F.P., Hoepner L.A., Herbstman J. (2020). Prenatal and early childhood exposure to phthalates and childhood behavior at age 7 years. Environ. Int..

[146] Zhang Q., Chen X.-Z., Huang X., Wang M., Wu J. (2019). The association between prenatal exposure to phthalates and cognition and neurobehavior of children-evidence from birth cohorts. Neurotoxicology.

[147] Engel S.M., Miodovnik A., Canfield R.L., Zhu C., Silva M.J., Calafat A.M., Wolff M.S., Engel S.M., Miodovnik A., Canfield R.L. (2010). Prenatal phthalate exposure is associated with childhood behavior and executive functioning. Environ. Health Perspect..

[148] Van den Dries M.A., Guxens M., Spaan S., Ferguson K.K., Philips E., Santos S., Jaddoe V.W., Ghassabian A., Trasande L., Tiemeier H. (2020). Phthalate and Bisphenol Exposure during Pregnancy and Offspring Nonverbal IQ. Environ. Health Perspect..

[149] Olesen T.S., Bleses D., Andersen H.R., Grandjean P., Frederiksen H., Trecca F., Bilenberg N., Kyhl H.B., Dalsager L., Jensen I.K. (2018). Prenatal phthalate exposure and language development in toddlers from the Odense Child Cohort. Neurotoxicol. Teratol..

[150] Ramhøj L., Frädrich C., Svingen T., Scholze M., Wirth E.K., Rijntjes E., Köhrle J., Kortenkamp A., Axelstad M. (2021). Testing for heterotopia formation in rats after developmental exposure to selected in vitro inhibitors of thyroperoxidase. Environ. Pollut..

[151] O’shaughnessy K.L., Kosian P.A., Ford J.L., Oshiro W.M., Degitz S.J., Gilbert M.E. (2018). Developmental Thyroid Hormone Insufficiency Induces a Cortical Brain Malformation and Learning Impairments: A Cross-Fostering Study. Toxicol. Sci..

[152] Crofton K.M., Gilbert M., Friedman K.P., Demeneix B., Marty M.S., Zoeller R.T. (2019). Adverse Outcome Pathway on Inhibition of Thyroperoxidase and Subsequent Adverse Neurodevelopmental Outcomes in Mammals.

[153] Gibson E.A., Siegel E.L., Eniola F., Herbstman J.B., Factor-Litvak P. (2018). Effects of Polybrominated Diphenyl Ethers on Child Cognitive, Behavioral, and Motor Development. Int. J. Environ. Res. Public Health.

[154] Lam J., Lanphear B.P., Bellinger D., Axelrad D.A., McPartland J., Sutton P., Davidson L., Daniels N., Sen S., Woodruff T.J. (2017). Developmental PBDE Exposure and IQ/ADHD in Childhood: A Systematic Review and Meta-analysis. Environ. Health Perspect..

[155] Vuong A.M., Yolton K., Xie C., Webster G.M., Sjödin A., Braun J.M., Dietrich K.N., Lanphear B.P., Chen A. (2017). Childhood polybrominated diphenyl ether (PBDE) exposure and neurobehavior in children at 8 years. Environ. Res..

[156] Herbstman J.B., Mall J.K. (2014). Developmental Exposure to Polybrominated Diphenyl Ethers and Neurodevelopment. Curr. Environ. Health Rep..

[157] Roze E., Meijer L., Bakker A., Van Braeckel K.N.J.A., Sauer P.J.J., Bos A.F. (2009). Prenatal exposure to organohalogens, including brominated flame retardants, influences motor, cognitive, and behavioral performance at school age. Environ. Health Perspect..

[158] Wang Y., Qian H. (2021). Phthalates and Their Impacts on Human Health. Healthcare.

[159] Berghuis S.A., Soechitram S.D., Hitzert M.M., Sauer P.J., Bos A.F. (2013). Prenatal exposure to polychlorinated biphenyls and their hydroxylated metabolites is associated with motor development of three-month-old infants. Neurotoxicology.

[160] Crofton K.M., Zoeller R.T. (2005). Mode of action: Neurotoxicity induced by thyroid hormone disruption during development--hearing loss resulting from exposure to PHAHs. Crit. Rev. Toxicol..

[161] Paul K.B., Hedge J.M., Bansal R., Zoeller R.T., Peter R., DeVito M., Crofton K.M. (2012). Developmental triclosan exposure decreases maternal, fetal, and early neonatal thyroxine: A dynamic and kinetic evaluation of a putative mode-of-action. Toxicology.

[162] Wang X., Ouyang F., Fengxiu O., Wang X., Liu Z., Zhang J. (2017). Maternal Urinary Triclosan Concentration in Relation to Maternal and Neonatal Thyroid Hormone Levels: A Prospective Study. Environ. Health Perspect..

[163] Johannes J., Jayarama-Naidu R., Meyer F., Wirth E.K., Schweizer U., Schomburg L., Köhrle J., Renko K. (2016). Silychristin, a Flavonolignan Derived from the Milk Thistle, Is a Potent Inhibitor of the Thyroid Hormone Transporter MCT8. Endocrinology.

[164] Ruel M.V.M., Bos A.F., Soechitram S.D., Meijer L., Sauer P.J.J., Berghuis S.A. (2019). Prenatal exposure to organohalogen compounds and children’s mental and motor development at 18 and 30 months of age. Neurotoxicology.

[165] Santoro A., Chianese R., Troisi J., Richards S., Nori S.L., Fasano S., Guida M., Plunk E., Viggiano A., Pierantoni R. (2019). Neuro-toxic and Reproductive Effects of BPA. Curr. Neuropharmacol..

[166] Kim M.J., Park Y.J. (2019). Bisphenols and Thyroid Hormone. Endocrinol. Metab..

[167] Khan M.A., Hansen L.G. (2003). Ortho-substituted polychlorinated biphenyl (PCB) congeners (95 or 101) decrease pituitary response to thyrotropin releasing hormone. Toxicol. Lett..

[168] Chen Y., Zhang W., Chen J., Wang N., Chen C., Wang Y., Wan H., Chen B., Lu Y. (2021). Association of Phthalate Exposure with Thyroid Function and Thyroid Homeostasis Parameters in Type 2 Diabetes. J. Diabetes Res..

[169] Wang Y., Hu C., Fang T., Jin Y., Wu R. (2021). Perspective on prenatal polychlorinated biphenyl exposure and the development of the progeny nervous system (Review). Int. J. Mol. Med..

[170] Abdelouahab N., Langlois M.-F., Lavoie L., Corbin F., Pasquier J.-C., Takser L. (2013). Maternal and cord-blood thyroid hormone levels and exposure to polybrominated diphenyl ethers and polychlorinated biphenyls during early pregnancy. Am. J. Epidemiol..

[171] Derakhshan A., Shu H., Peeters R.P., Kortenkamp A., Lindh C.H., Demeneix B., Bornehag C.-G., Korevaar T.I.M. (2019). Association of urinary bisphenols and triclosan with thyroid function during early pregnancy. Environ. Int..

[172] Manikkam M., Tracey R., Guerrero-Bosagna C., Skinner M.K. (2013). Plastics derived endocrine disruptors (BPA, DEHP and DBP) induce epigenetic transgenerational inheritance of obesity, reproductive disease and sperm epimutations. PLoS ONE.

[173] Hernandez A., Stohn J.P. (2018). The Type 3 Deiodinase: Epigenetic Control of Brain Thyroid Hormone Action and Neurological Function. Int. J. Mol. Sci..

[174] Kyono Y., Subramani A., Ramadoss P., Hollenberg A.N., Bonett R.M., Denver R.J. (2016). Liganded Thyroid Hormone Receptors Transactivate the DNA Methyltransferase 3a Gene in Mouse Neuronal Cells. Endocrinology.

[175] Walker C.L. (2016). Minireview: Epigenomic Plasticity and Vulnerability to EDC Exposures. Mol. Endocrinol..

[176] Pitto L., Gorini F., Bianchi F., Guzzolino E. (2020). New Insights into Mechanisms of Endocrine-Disrupting Chemicals in Thyroid Diseases: The Epigenetic Way. Int. J. Environ. Res. Public Health.

[177] Alavian-Ghavanini A., Rüegg J. (2018). Understanding Epigenetic Effects of Endocrine Disrupting Chemicals: From Mechanisms to Novel Test Methods. Basic Clin. Pharmacol. Toxicol..

[178] Poston R.G., Saha R.N. (2019). Epigenetic Effects of Polybrominated Diphenyl Ethers on Human Health. Int. J. Environ. Res. Public Health.

[179] Hay I., Hynes K.L., Burgess J.R. (2019). Mild-to-Moderate Gestational Iodine Deficiency Processing Disorder. Nutrients.

[180] Thompson W., Russell G., Baragwanath G., Matthews J., Vaidya B., Thompson-Coon J. (2018). Maternal thyroid hormone insufficiency during pregnancy and risk of neurodevelopmental disorders in offspring: A systematic review and meta-analysis. Clin. Endocrinol..

[181] Pop V.J., Brouwers E.P., Vader H.L., Vulsma T., Van Baar A.L., De Vijlder J.J. (2003). Maternal hypothyroxinaemia during early pregnancy and subsequent child development: A 3-year follow-up study. Clin. Endocrinol..

[182] Li Y., Shan Z., Teng W., Yu X., Li Y., Fan C., Teng X., Guo R., Wang H., Li J. (2010). Abnormalities of maternal thyroid function during pregnancy affect neuropsychological development of their children at 25–30 months. Clin. Endocrinol..

[183] Henrichs J., Bongers-Schokking J.J., Schenk J.J., Ghassabian A., Schmidt H.G., Visser T.J., Hooijkaas H., de Muinck Keizer-Schrama S.M., Hofman A., Jaddoe V.V. (2010). Maternal thyroid function during early pregnancy and cognitive functioning in early childhood: The generation R study. J. Clin. Endocrinol. Metab..

[184] Williams F., Watson J., Ogston S., Hume R., Willatts P., Visser T., Scottish Preterm Thyroid Group (2012). Mild maternal thyroid dysfunction at delivery of infants born ≤34 weeks and neurodevelopmental outcome at 5.5 years. J. Clin. Endocrinol. Metab..

[185] Craig W.Y., Allan W.C., Kloza E.M., Pulkkinen A.J., Waisbren S., Spratt D.I., Palomaki G.E., Neveux L.M., Haddow J.E. (2012). Mid-gestational maternal free thyroxine concentration and offspring neurocognitive development at age two years. J. Clin. Endocrinol. Metab..

[186] Päkkilä F., Männistö T., Hartikainen A.-L., Ruokonen A., Surcel H.-M., Bloigu A., Vääräsmäki M., Järvelin M.-R., Moilanen I., Suvanto E. (2015). Maternal and Child’s Thyroid Function and Child’s Intellect and Scholastic Performance. Thyroid.

[187] Grau G., Aguayo A., Vela A., Aniel-Quiroga A., Espada M., Miranda G., Martinez-Indart L., Martul P., Castaño L., Rica I. (2015). Normal intellectual development in children born from women with hypothyroxinemia during their pregnancy. J. Trace Elem. Med. Biol..

[188] Noten A.M., Loomans E.M., Vrijkotte T.G.M., van de Ven P.M., van Trotsenburg A.S.P., Rotteveel J., van Eijsden M., Finken M.J.J. (2015). Maternal hypothyroxinaemia in early pregnancy and school performance in 5-year-old offspring. Eur. J. Endocrinol..

[189] Kampouri M., Margetaki K., Koutra K., Kyriklaki A., Karakosta P., Anousaki D., Chalkiadaki G., Vafeiadi M., Kogevinas M., Chatzi L. (2021). Maternal mild thyroid dysfunction and offspring cognitive and motor development from infancy to childhood: The Rhea mother-child cohort study in Crete, Greece. J. Epidemiol. Community Health.

[190] Andersen S.L., Andersen S., Liew Z., Vestergaard P., Olsen J. (2018). Maternal Thyroid Function in Early Pregnancy and Neuropsychological Performance of the Child at 5 Years of Age. J. Clin. Endocrinol. Metab..

[191] Chevrier J., Harley K.G., Kogut K., Holland N., Johnson C., Eskenazi B. (2011). Maternal Thyroid Function during the Second Half of Pregnancy and Child Neurodevelopment at 6, 12, 24, and 60 Months of Age. J. Thyroid Res..

[192] Fetene D.M., Betts K.S., Alati R. (2017). Mechanisms in endocrinology: Maternal thyroid dysfunction during pregnancy and behavioural and psychiatric disorders of children: A systematic review. Eur. J. Endocrinol..

[193] Drover S.S.M., Villanger G.D., Aase H., Skogheim T.S., Longnecker M.P., Zoeller R.T., Reichborn-Kjennerud T., Knudsen G.P., Zeiner P., Engel S.M. (2019). Maternal Thyroid Function During Pregnancy or Neonatal Thyroid Function and Attention Deficit Hyperactivity Disorder: A Systematic Review. Epidemiology.

[194] Fan X., Wu L. (2016). The impact of thyroid abnormalities during pregnancy on subsequent neuropsychological development of the offspring: A meta-analysis. J. Matern. Fetal Neonatal Med..

[195] Wang P., Gao J., Zhao S., Guo Y., Wang Z., Qi F. (2016). Maternal Thyroxine Levels During Pregnancy and Outcomes of Cognitive Development in Children. Mol. Neurobiol..

[196] Levie D., Korevaar T.I.M., Bath S.C., Murcia M., Dineva M., Llop S., Espada M., Van Herwaarden A.E., De Rijke Y.B., Ibarluzea J.M. (2019). Association of Maternal Iodine Status with Child IQ: A Meta-Analysis of Individual Participant Data. J. Clin. Endocrinol. Metab..

[197] Levie D., Korevaar T.I.M., Bath S., Dalmau-Bueno A., Murcia M., Espada M., Dineva M., Ibarluzea J.M., Sunyer J., Tiemeier H. (2018). Thyroid Function in Early Pregnancy, Child IQ and Autistic Traits: A Meta-Analysis of Individual Participant Data. J. Clin. Endocrinol. Metab..

[198] Leemans M., Couderq S., Demeneix B., Fini J.-B. (2019). Pesticides with Potential Thyroid Hormone-Disrupting Effects: A Review of Recent Data. Front. Endocrinol..

[199] Grova N., Schroeder H., Olivier J.L., Turner J.D. (2019). Epigenetic and Neurological Impairments Associated with Early Life Exposure to Persistent Organic Pollutants. Int. J. Genom..

[200] Lenters V., Iszatt N., Forns J., Čechová E., Kočan A., Legler J., Leonards P., Stigum H., Eggesbø M. (2019). Early-life exposure to persistent organic pollutants (OCPs, PBDEs, PCBs, PFASs) and attention-deficit/hyperactivity disorder: A multi-pollutant analysis of a Norwegian birth cohort. Environ. Int..

[201] Rahman M., Shu Y.-H., Chow T., Lurmann F.W., Yu X., Martinez M.P., Carter S.A., Eckel S.P., Chen J.-C., Chen Z. (2022). Prenatal Exposure to Air Pollution and Autism Spectrum Disorder: Sensitive Windows of Exposure and Sex Differences. Environ. Health Perspect..

[202] Vermiglio F., Presti V.P.L., Moleti M., Sidoti M., Tortorella G., Scaffidi G., Castagna M.G., Mattina F., Violi M.A., Crisà A. (2004). Attention deficit and hyperactivity disorders in the offspring of mothers exposed to mild-moderate iodine deficiency: A possible novel iodine deficiency disorder in developed countries. J. Clin. Endocrinol. Metab..

[203] Gaberšček S., Zaletel K. (2016). Epidemiological trends of iodine-related thyroid disorders: An example from Slovenia. Arh. Hig. Rada Toksikol..

[204] Román G.C. (2007). Autism: Transient in utero hypothyroxinemia related to maternal flavonoid ingestion during pregnancy and to other environmental antithyroid agents. J. Neurol. Sci..

[205] Lewandowski T.A., Peterson M.K., Charnley G. (2015). Iodine supplementation and drinking-water perchlorate mitigation. Food Chem. Toxicol..

[206] Leung A.M., Pearce E.N., Braverman L.E. (2014). Environmental perchlorate exposure: Potential adverse thyroid effects. Curr Opin Endocrinol. Diabetes Obes..

[207] Charatcharoenwitthaya N., Ongphiphadhanakul B., Pearce E.N., Somprasit C., Chanthasenanont A., He X., Chailurkit L., Braverman L.E. (2014). The association between perchlorate and thiocyanate exposure and thyroid function in first-trimester pregnant Thai women. J. Clin. Endocrinol. Metab..

[208] Ozpinar A., Kelestimur F., Songur Y., Can O., Valentin L., Caldwell K., Arikan E., Ünsal I., Serteser M., Inal T. (2014). Iodine status in Turkish populations and exposure to iodide uptake inhibitors. PLoS ONE.

[209] Suh M., Abraham L., Hixon J.G., Proctor D.M. (2014). The effects of perchlorate, nitrate, and thiocyanate on free thyroxine for potentially sensitive subpopulations of the 2001–2002 and 2007–2008 National Health and Nutrition Examination Surveys. J. Expo. Sci. Environ. Epidemiol..

[210] Lumen A., Mattie D.R., Fisher J.W. (2013). Evaluation of perturbations in serum thyroid hormones during human pregnancy due to dietary iodide and perchlorate exposure using a biologically based dose-response model. Toxicol. Sci..

[211] Breous E., Wenzel A., Loos U. (2005). The promoter of the human sodium/iodide symporter responds to certain phthalate plasticisers. Mol. Cell. Endocrinol..

[212] Liu C., Zhao L., Wei L., Li L. (2015). DEHP reduces thyroid hormones via interacting with hormone synthesis-related proteins, deiodinases, transthyretin, receptors, and hepatic enzymes in rats. Environ. Sci. Pollut. Res. Int..

[213] Villanger G.D., Drover S.S., Nethery R.C., Thomsen C., Sakhi A.K., Øvergaard K.R., Zeiner P., Hoppin J., Reichborn-Kjennerud T., Aase H. (2020). Associations between urine phthalate metabolites and thyroid function in pregnant women and the influence of iodine status. Environ. Int..

[214] Kamai E.M., Villanger G.D., Nethery R.C., Thomsen C., Sakhi A.K., Drover S.S.M., Hoppin J.A., Knudsen G.P., Reichborn-Kjennerud T., Zeiner P. (2021). Gestational Phthalate Exposure and Preschool Attention Deficit Hyperactivity Disorder in Norway. Environ. Epidemiol..

[215] Merced-Nieves F.M., Dzwilewski K.L.C., Aguiar A., Musaad S., Korrick S.A., Schantz S.L. (2021). Associations of Prenatal Exposure to Phthalates with Measures of Cognition in 4.5-Month-Old Infants. Int. J. Environ. Res. Public Health.

[216] Jankowska A., Nazareth L., Kaleta D., Polanska K. (2021). Review of the Existing Evidence for Sex-Specific Relationships between Prenatal Phthalate Exposure and Children’s Neurodevelopment. Int. J. Environ. Res. Public Health.

[217] Lucaccioni L., Trevisani V., Passini E., Righi B., Plessi C., Predieri B., Iughetti L. (2021). Perinatal Exposure to Phthalates: From Endocrine to Neurodevelopment Effects. Int. J. Mol. Sci..

[218] Romano M.E., Webster G.M., Vuong A.M., Zoeller R.T., Chen A., Hoofnagle A.N., Calafat A.M., Karagas M.R., Yolton K., Lanphear B.P. (2015). Gestational urinary bisphenol A and maternal and newborn thyroid hormone concentrations: The HOME Study. Environ. Res..

[219] Wang X., Tang N., Nakayama S.F., Fan P., Liu Z., Zhang J., Ouyang F. (2020). Maternal urinary bisphenol A concentration and thyroid hormone levels of Chinese mothers and newborns by maternal body mass index. Environ. Sci. Pollut. Res. Int..

[220] Li F., Yang F., Li D.-K., Tian Y., Miao M., Zhang Y., Ji H., Yuan W., Liang H. (2020). Prenatal bisphenol A exposure, fetal thyroid hormones and neurobehavioral development in children at 2 and 4 years: A prospective cohort study. Sci. Total Environ..

[221] Braun J.M., Yolton K., Stacy S.L., Erar B., Papandonatos G.D., Bellinger D.C., Lanphear B.P., Chen A. (2017). Prenatal environmental chemical exposures and longitudinal patterns of child neurobehavior. Neurotoxicology.

[222] Lim Y.-H., Bae S., Kim B.-N., Shin C.H., Lee Y.A., Kim J.I., Hong Y.-C. (2017). Prenatal and postnatal bisphenol A exposure and social impairment in 4-year-old children. Environ. Health.

[223] Ejaredar M., Lee Y., Roberts D.J., Sauve R., Dewey D. (2017). Bisphenol A exposure and children’s behavior: A systematic review. J. Expo. Sci. Environ. Epidemiol..

[224] Koutaki D., Paltoglou G., Vourdoumpa A., Charmandari E. (2021). The Impact of Bisphenol A on Thyroid Function in Neonates and Children: A Systematic Review of the Literature. Nutrients.

[225] Minatoya M., Kishi R. (2021). A Review of Recent Studies on Bisphenol A and Phthalate Exposures and Child Neurodevelopment. Int. J. Environ. Res. Public Health.

[226] Brucker-Davis F., Ganier-Chauliac F., Gal J., Panaïa-Ferrari P., Pacini P., Fénichel P., Hiéronimus S. (2015). Neurotoxicant exposure during pregnancy is a confounder for assessment of iodine supplementation on neurodevelopment outcome. Neurotoxicol. Teratol..

[227] Torres-Sánchez L., Gamboa R., Bassol-Mayagoitia S., Huesca-Gómez C., Nava M.P., Vázquez-Potisek J.I., Yáñez-Estrada L., Mejía-Saucedo R., Blanco-Muñoz J. (2019). Para-occupational exposure to pesticides, PON1 polymorphisms and hypothyroxinemia during the first half of pregnancy in women living in a Mexican floricultural area. Environ. Health.

[228] Eskenazi B., An S., Rauch S.A., Coker E.S., Maphula A., Obida M., Crause M., Kogut K.R., Bornman R., Chevrier J. (2018). Prenatal Exposure to DDT and Pyrethroids for Malaria Control and Child Neurodevelopment: The VHEMBE Cohort, South Africa. Environ. Health Perspect..

[229] Chevrier J., Rauch S., Obida M., Crause M., Bornman R., Eskenazi B. (2019). Sex and poverty modify associations between maternal peripartum concentrations of DDT/E and pyrethroid metabolites and thyroid hormone levels in neonates participating in the VHEMBE study, South Africa. Environ. Int..

[230] Steinmaus C., Pearl M., Kharrazi M., Blount B.C., Miller M.D., Pearce E.N., Valentin-Blasini L., DeLorenze G., Hoofnagle A.N., Liaw J. (2016). Thyroid Hormones and Moderate Exposure to Perchlorate during Pregnancy in Women in Southern California. Environ. Health Perspect..

[231] Cowell W.J., Sjödin A., Jones R., Wang Y., Wang S., Whyatt R.M., Factor-Litvak P., Bradwin G., Hassoun A., Oberfield S. (2019). Pre- and Postnatal Polybrominated Diphenyl Ether Concentrations in Relation to Thyroid Parameters Measured During Early Childhood. Thyroid.

[232] Jedynak P., Maitre L., Guxens M., Gützkow K.B., Julvez J., López-Vicente M., Sunyer J., Casas M., Chatzi L., Gražulevičienė R. (2021). Prenatal exposure to a wide range of environmental chemicals and child behaviour between 3 and 7 years of age—An exposome-based approach in 5 European cohorts. Sci. Total Environ..

[233] Leko M.B., Gunjača I., Pleić N., Zemunik T. (2021). Environmental Factors Affecting Thyroid-Stimulating Hormone and Thyroid Hormone Levels. Int. J. Mol. Sci..

[234] Ho V., Pelland-St-Pierre L., Gravel S., Bouchard M., Verner M.-A., Labrèche F. (2022). Endocrine disruptors: Challenges and future directions in epidemiologic research. Environ. Res..

[235] Salazar P., Villaseca P., Cisternas P., Inestrosa N.C. (2021). Neurodevelopmental impact of the offspring by thyroid hormone system-disrupting environmental chemicals during pregnancy. Environ. Res..

[236] Gheidarloo M., Kelishadi R., Hovsepian S., Keikha M., Hashemipour M. (2020). The association between prenatal exposure to organochlorine compounds and neonatal thyroid hormone levels: A systematic review. J. Pediatr. Endocrinol. Metab..

[237] World Health Organization (2021). WHO Global Air Quality Guidelines: Particulate Matter (PM2.5 and PM10), Ozone, Nitrogen Dioxide, Sulfur Dioxide and Carbon Monoxide: Executive Summary.

[238] WHO (2021). Fact Sheets 22 September 2021 Ambient (Outdoor) Air Quality and Health.

[239] Burnett R., Chen H., Szyszkowicz M., Fann N., Hubbell B., Pope C.A., Apte J.S., Brauer M., Cohen A., Weichenthal S. (2018). Global estimates of mortality associated with long-term exposure to outdoor fine particulate matter. Proc. Natl. Acad. Sci. USA.

[240] Marty M.A., Perera F., Miller M.D., Swanson M., Ellickson K., Cory-Slechta D.A., Ritz B., Balmes J., Anderko L., Talbott E.O. (2019). Healthy Air, Healthy Brains: Advancing Air Pollution Policy to Protect Children’s Health. Am. J. Public Health.

[241] Loftus C.T., Ni Y., Szpiro A.A., Hazlehurst M.F., Tylavsky F.A., Bush N.R., Sathyanarayana S., Carroll K.N., Young M., Karr C.J. (2020). Exposure to ambient air pollution and early childhood behavior: A longitudinal cohort study. Environ. Res..

[242] Peterson B.S., Rauh V.A., Bansal R., Hao X., Toth Z., Nati G., Walsh K., Miller R.L., Arias F., Semanek D. (2015). Effects of prenatal exposure to air pollutants (polycyclic aromatic hydrocarbons) on the development of brain white matter, cognition, and behavior in later childhood. JAMA Psychiatry.

[243] Zhao Y., Cao Z., Li H., Su X., Yang Y., Liu C., Hua J. (2019). Air pollution exposure in association with maternal thyroid function during early pregnancy. J. Hazard. Mater..

[244] Charoenratana C., Leelapat P., Traisrisilp K., Tongsong T. (2016). Maternal iodine insufficiency and adverse pregnancy outcomes. Matern. Child Nutr..

[245] Holland E.B., Pessah I.N. (2021). Non-dioxin-like polychlorinated biphenyl neurotoxic equivalents found in environmental and human samples. Regul. Toxicol. Pharmacol..

[246] Block M.L., Elder A., Auten R.L., Bilbo S.D., Chen H., Chen J.-C., Cory-Slechta D.A., Costa D., Diaz-Sanchez D., Dorman D.C. (2012). The outdoor air pollution and brain health workshop. Neurotoxicology.

[247] Lubczyńska M.J., Muetzel R.L., El Marroun H., Basagaña X., Strak M., Denault W., Jaddoe V.W., Hillegers M., Vernooij M.W., Hoek G. (2020). Exposure to Air Pollution during Pregnancy and Childhood, and White Matter Microstructure in Preadolescents. Environ. Health Perspect..

[248] Guxens M., Lubczyńska M.J., Muetzel R.L., Dalmau-Bueno A., Jaddoe V.W., Hoek G., van der Lugt A., Verhulst F.C., White T., Brunekreef B. (2018). Air Pollution Exposure During Fetal Life, Brain Morphology, and Cognitive Function in School-Age Children. Biol. Psychiatry.

[249] Guxens M., Garcia-Esteban R., Giorgis-Allemand L., Forns J., Badaloni C., Ballester F., Beelen R., Cesaroni G., Chatzi L., de Agostini M. (2014). Air pollution during pregnancy and childhood cognitive and psychomotor development: Six European birth cohorts. Epidemiology.

[250] Kim E., Park H., Hong Y.-C., Ha M., Kim Y., Kim B.-N., Kim Y., Roh Y.-M., Lee B.-E., Ryu J.-M. (2014). Prenatal exposure to PM_10_ and NO_2_ and children’s neurodevelopment from birth to 24 months of age: Mothers and Children’s Environmental Health (MOCEH) study. Sci. Total Environ..

[251] Calderón-Garcidueñas L., Mora-Tiscareño A., Franco-Lira M., Zhu H., Lu Z., Solorio E., Torres-Jardón R., D’Angiulli A. (2015). Decreases in Short Term Memory, IQ, and Altered Brain Metabolic Ratios in Urban Apolipoprotein ε4 Children Exposed to Air Pollution. J. Alzheimer’s Dis..

[252] Chiu Y.-H.M., Hsu H.-H.L., Coull B.A., Bellinger D.C., Kloog I., Schwartz J., Wright R.O., Wright R.J. (2016). Prenatal particulate air pollution and neurodevelopment in urban children: Examining sensitive windows and sex-specific associations. Environ. Int..

[253] Lam J., Sutton P., Kalkbrenner A., Windham G., Halladay A., Koustas E., Lawler C., Davidson L., Daniels N., Newschaffer C. (2016). A Systematic Review and Meta-Analysis of Multiple Airborne Pollutants and Autism Spectrum Disorder. PLoS ONE.

[254] Kalkbrenner A.E., Windham G.C., Serre M.L., Akita Y., Wang X., Hoffman K., Thayer B.P., Daniels J.L. (2015). Particulate matter exposure, prenatal and postnatal windows of susceptibility, and autism spectrum disorders. Epidemiology.

[255] Volk H.E., Lurmann F., Penfold B., Hertz-Picciotto I., McConnell R. (2013). Traffic-related air pollution, particulate matter, and autism. JAMA Psychiatry.

[256] Clifford A., Lang L., Chen R., Anstey K.J., Seaton A. (2016). Exposure to air pollution and cognitive functioning across the life course—A systematic literature review. Environ. Res..

[257] Gong T., Dalman C., Wicks S., Dal H., Magnusson C., Lundholm C., Almqvist C., Pershagen G. (2017). Perinatal Exposure to Traffic-Related Air Pollution and Autism Spectrum Disorders. Environ. Health Perspect..

[258] Luminati O., Brentani A., Flückiger B., Ledebur de Antas de Campos B., Raess M., Röösli M., de Hoogh K., Fink G. (2022). Assessing the association between air pollution and child development in São Paulo, Brazil. PLoS ONE.

[259] Lee S.Y., Pearce E.N. (2021). Testing, Monitoring, and Treatment of Thyroid Dysfunction in Pregnancy. J. Clin. Endocrinol. Metab..

[260] Leung A.M. (2018). No Benefit of Levothyroxine Among Pregnant Hypothyroid and/or Hypothyroxinemic Women on Offspring IQ at Age 9 years. Clin. Thyroidol..

[261] Runkle I., de Miguel M.P., Barabash A., Cuesta M., Diaz Á., Duran A., Familiar C., de la Torre N.G., Herraiz M.Á., Izquierdo N. (2021). Early Levothyroxine Treatment for Subclinical Hypothyroidism or Hypothyroxinemia in Pregnancy: The St Carlos Gestational and Thyroid Protocol. Front. Endocrinol..

[262] Young A.E., Kemp J.F., Uhlson C., Westcott J.L., Ali S.A., Saleem S., Garcès A., Figueroa L., Somannavar M.S., Goudar S.S. (2021). Women First Preconception Maternal Nutrition Trial Group. Improved first trimester maternal iodine status with preconception supplementation: The Women First Trial. Matern. Child Nutr..

[263] Berbel P., Mestre J.L., Santamaría A., Palazón I., Franco A., Graells M., González-Torga A., De Escobar G.M. (2009). Delayed neurobehavioral development in children born to pregnant women with mild hypothyroxinemia during the first month of gestation: The importance of early iodine supplementation. Thyroid.

[264] Dineva M., Fishpool H., Rayman M.P., Mendis J., Bath S.C. (2020). Systematic review and meta-analysis of the effects of iodine supplementation on thyroid function and child neurodevelopment in mildly-to-moderately iodine-deficient pregnant women. Am. J. Clin. Nutr..

[265] Robinson S.M., Crozier S.R., Miles E.A., Gale C.R., Calder P.C., Cooper C., Inskip H.M., Godfrey K.M. (2018). Preconception Maternal Iodine Status Is Positively Associated with IQ but Not with Measures of Executive Function in Childhood. J. Nutr..

[266] Moleti M., Presti V.P.L., Campolo M.C., Mattina F., Galletti M., Mandolfino M., Violi M.A., Giorgianni G., De Domenico D., Trimarchi F. (2008). Iodine prophylaxis using iodized salt and risk of maternal thyroid failure in conditions of mild iodine deficiency. J. Clin. Endocrinol. Metab..

[267] Nussey S., Whitehead S. (2001). Endocrinology: An Integrated Approach.

[268] Hynes K.L., Seal J.A., Otahal P., Oddy W.H., Burgess J.R. (2019). Women Remain at Risk of Iodine Deficiency during Pregnancy: The Importance of Iodine Supplementation before Conception and Throughout Gestation. Nutrients.

[269] Machamba A.A.L., Azevedo F.M., Fracalossi K.O., Franceschini S.D.C.C. (2021). Effect of iodine supplementation in pregnancy on neurocognitive development on offspring in iodine deficiency areas: A systematic review. Arch. Endocrinol. Metab..

[270] Nazeri P., Shariat M., Azizi F. (2021). Effects of iodine supplementation during pregnancy on pregnant women and their offspring: A systematic review and meta-analysis of trials over the past 3 decades. Eur. J. Endocrinol..

[271] Harding K.B., Peña-Rosas J.P., Webster A.C., Yap C.M.Y., Payne B.A., Ota E., De-Regil L.M. (2017). Iodine supplementation for women during the preconception, pregnancy and postpartum period. Cochrane Database Syst. Rev..

[272] Verhagen N.J.E., Gowachirapant S., Winichagoon P., Andersson M., Melse-Boonstra A., Zimmermann M.B. (2020). Iodine Supplementation in Mildly Iodine-Deficient Pregnant Women Does Not Improve Maternal Thyroid Function or Child Development: A Secondary Analysis of a Randomized Controlled Trial. Front. Endocrinol..

[273] Abel M.H., Korevaar T.I., Erlund I., Villanger G.D., Caspersen I.H., Arohonka P., Alexander J., Meltzer H.M., Brantsaeter A.L. (2018). Iodine Intake is Associated with Thyroid Function in Mild to Moderately Iodine Deficient Pregnant Women. Thyroid.

[274] Pearce E.N., Lazarus J.H., Moreno-Reyes R., Zimmermann M.B. (2016). Consequences of iodine deficiency and excess in pregnant women: An overview of current knowns and unknowns. Am. J. Clin. Nutr..

[275] Manousou S., Eggertsen R., Hulthén L., Nyström H.F. (2021). A randomized, double-blind study of iodine supplementation during pregnancy in Sweden: Pilot evaluation of maternal iodine status and thyroid function. Eur. J. Nutr..

[276] Lopes-Pereira M., Roque S., Costa P., Quialheiro A., Santos N.C., Goios A., Vilarinho L., Correia-Neves M., Palha J.A. (2020). Impact of iodine supplementation during preconception, pregnancy and lactation on maternal thyroid homeostasis and offspring psychomotor development: Protocol of the IodineMinho prospective study. BMC Pregnancy Childbirth.

[277] Bell M.A., Ross A.P., Goodman G. (2016). Assessing infant cognitive development after prenatal iodine supplementation. Am. J. Clin. Nutr..

[278] Troendle J.F. (2016). Statistical design considerations applicable to clinical trials of iodine supplementation in pregnant women who may be mildly iodine deficient. Am. J. Clin. Nutr..

[279] Shi X., Han C., Li C., Mao J., Wang W., Xie X., Li C., Xu B., Meng T., Du J. (2015). Optimal and safe upper limits of iodine intake for early pregnancy in iodine-sufficient regions: A cross-sectional study of 7190 pregnant women in China. J. Clin. Endocrinol. Metab..

[280] Schaffner M., Mühlberger N., Conrads-Frank A., Rushaj V.Q., Sroczynski G., Koukkou E., Thuesen B.H., Völzke H., Oberaigner W., Siebert U. (2021). Benefits and Harms of a Prevention Program for Iodine Deficiency Disorders: Predictions of the Decision-Analytic EUthyroid Model. Thyroid.

